# 24-hour diastolic blood pressure average real variability predicts renal progression in diabetic kidney disease: a comprehensive cohort study

**DOI:** 10.3389/fendo.2026.1834470

**Published:** 2026-04-20

**Authors:** Yanqing Hu, Shan Ma, Ting Yu, Dan Wu, Yang Wu

**Affiliations:** 1 Department of Orthopedics, The Seventh Affiliated Hospital, Sun Yat-sen University, Shenzhen, China; 2 Department of Cardiology, The Seventh Affiliated Hospital, Sun Yat-sen University, Shenzhen, China; 3 Department of Endocrinology, The Seventh Affiliated Hospital, Sun Yat-sen University, Shenzhen, China; 4 Department of Emergency and Disaster Medical Center, The Seventh Affiliated Hospital, Sun Yat-sen University, Shenzhen, China

**Keywords:** ambulatory blood pressure monitoring, average real variability, diabetic kidney disease, diastolic blood pressure variability, end-stage renal disease, predictive modeling, renal function decline

## Abstract

**Objective:**

Diabetic kidney disease (DKD) is the leading cause of end-stage renal disease (ESRD) worldwide. Blood pressure variability (BPV), especially diastolic BPV (DBPV), is closely linked to renal microcirculation but remains understudied in DKD. This study aimed to evaluate the association of DBPV with renal progression, identify an optimal risk threshold, and explore antihypertensive drug implications.

**Methods:**

We conducted a retrospective cohort study of 2,143 DKD patients who underwent 24-hour ambulatory BP monitoring (ABPM) between 2018 and 2022, with a median follow-up of 4.8 years. Multiple DBPV parameters including standard deviation (SD), coefficient of variation (CV), average real variability (ARV), and nocturnal dipping were analyzed. Dynamic changes in DBPV were assessed in 1,328 patients with serial ABPM data.

**Results:**

After full adjustment, 24-hour DBP ARV was the strongest predictor of renal outcomes. Each 1 mmHg increase was associated with 18% higher odds of rapid estimated glomerular filtration rate (eGFR) decline (OR = 1.18, 95%CI:1.13–1.23), 22% higher ESRD risk (HR = 1.22, 95%CI:1.15–1.29), and 20% higher composite renal event risk (HR = 1.20, 95%CI:1.14–1.26). ROC analysis determined the optimal threshold of 24-hour DBP ARV for ESRD prediction as 10.2 mmHg (sensitivity=76.2%, specificity=61.8%), above which ESRD risk increased 3.1-fold. Patients with increased DBPV over time had a 2.4-fold higher ESRD risk than those with decreased DBPV. Calcium channel blockers (CCBs) were associated with lower DBP ARV than RAAS inhibitors or beta-blockers. Adding 24-hour DBP ARV to traditional risk models significantly improved ESRD prediction (C-statistic: 0.73 to 0.80). The association was stronger in patients with advanced DKD or severely increased albuminuria, and combined high DBP ARV and SBP ARV conferred a 4.5-fold higher ESRD risk.

**Conclusion:**

24-hour DBP ARV (threshold 10.2 mmHg) is an independent predictor of renal progression in DKD. Rising DBPV amplifies renal risk, and CCBs may better reduce DBPV. Incorporating ABPM-derived DBPV into DKD management improves risk stratification and supports personalized interventions.

## 1 Introduction

Diabetic kidney disease (DKD) is one of the most severe microvascular complications of type 1 and type 2 diabetes, affecting 20–40% of diabetic patients worldwide and accounting for over 50% of incident end-stage renal disease (ESRD) cases in high-income countries (1). Despite advances in glucose-lowering therapy (e.g., sodium-glucose cotransporter-2 inhibitors) and blood pressure (BP) control, the incidence of ESRD in DKD patients remains high, highlighting critical gaps in risk stratification and disease management (2, 3). Blood pressure control is a cornerstone of DKD management per the 2022 KDIGO Clinical Practice Guideline, which recommends a systolic BP target of <130 mmHg, but traditional BP metrics (e.g., office BP, mean 24-hour BP) fail to capture blood pressure variability (BPV)—the short-term fluctuations in BP over time (3, 4). Mounting evidence has identified BPV as an independent risk factor for cardiovascular and renal outcomes in hypertension and chronic kidney disease (CKD), but most studies have focused on systolic BPV (SBPV), with diastolic BPV (DBPV) remaining largely unexplored in DKD (5, 6).

Diastolic BP (DBP) is a key determinant of renal microcirculatory perfusion, as it reflects vascular resistance during ventricular relaxation and directly modulates intraglomerular pressure and renal blood flow (7, 8). In DKD, structural and functional renal vascular damage (e.g., arteriolar hyalinosis, endothelial dysfunction) impairs the myogenic autoregulatory response, rendering the kidney highly vulnerable to DBP fluctuations: transient DBP spikes increase intraglomerular pressure and induce podocyte injury, while nocturnal DBP dips reduce renal perfusion and trigger ischemic tubular damage (8, 9). Average real variability (ARV)—a dynamic DBPV parameter that quantifies the average of absolute differences between consecutive BP measurements—has been shown to better capture BP-induced hemodynamic insult to target organs than static metrics (e.g., standard deviation, coefficient of variation) in hypertension (10, 11), but its predictive value in DKD has not been evaluated. Additionally, abnormal nocturnal DBP dipping patterns and dynamic changes in DBPV over time are understudied in DKD, and the association between different antihypertensive medication classes and DBPV remains unclear in this patient population (12, 13).

This single-center retrospective cohort study addressed these knowledge gaps by comprehensively evaluating the association between multiple DBPV parameters (ARV, standard deviation, coefficient of variation, nocturnal dipping) and renal outcomes in a large DKD population. We aimed to: (1) identify the most predictive DBPV parameter for renal progression; (2) derive an internal hypothesis-generating DBPV threshold for ESRD risk stratification; (3) explore the association between dynamic DBPV changes and renal outcomes; and (4) compare DBPV parameters across different antihypertensive medication classes in this cohort. We also assessed the incremental predictive value of DBPV beyond traditional risk factors and evaluated effect modification by DKD stage and albuminuria status. The findings of this study provide hypothesis-generating evidence for the potential role of DBPV in DKD risk stratification, with all conclusions requiring validation in external multi-center cohorts.

## 2 Methods

### 2.1 Study population

**Figure 1:**
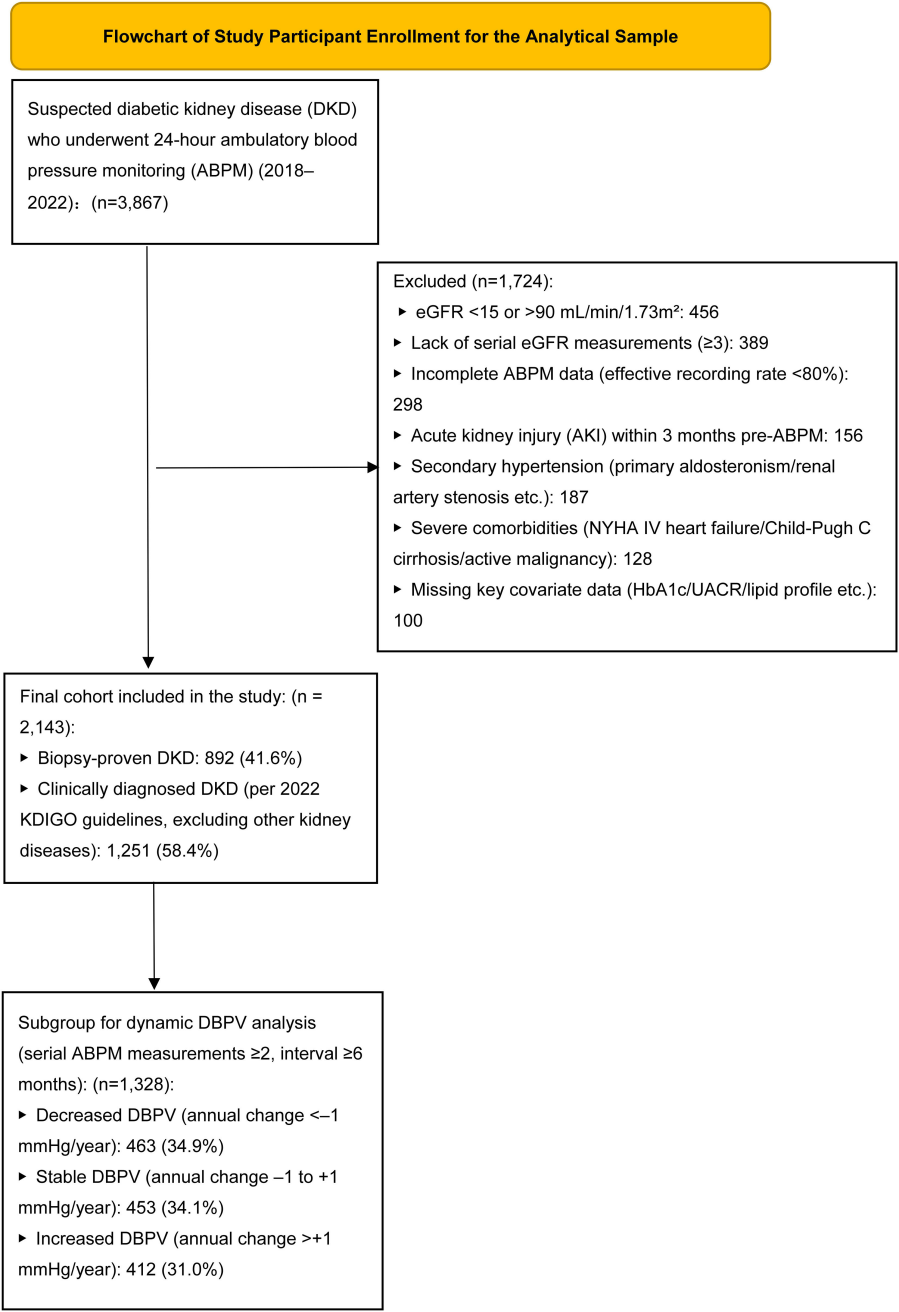
CONSORT-compliant patient flowchart.Screening, exclusion, enrollment, and stratification of the study population (2018–2022). Of 3,867 suspected DKD patients with 24-hour ABPM, 1,724 were excluded (abnormal eGFR, incomplete data, comorbidities, etc.). The final cohort (n=2,143) included biopsy-proven (41.6%) and clinically diagnosed (58.4%) DKD patients; 1,328 with serial ABPM were stratified by annual DBPV changes. All patients completed 4.8-year median follow-up.

This retrospective cohort study included patients with DKD from the Seventh Affiliated Hospital of Sun Yat-sen University (Shenzhen, China) who underwent 24-hour ABPM between January 2018 and December 2022, with a minimum follow-up duration of 12 months. A CONSORT-compliant patient flowchart (**Figure 1**) details the screening, exclusion, and final enrollment process: a total of 3,867 patients with suspected DKD underwent 24-hour ABPM during the study period; 1,724 patients were excluded for the following reasons: eGFR <15 or >90 mL/min/1.73m^2^ (n=456), lack of serial eGFR measurements (n=389), incomplete ABPM data (effective recording rate <80%, n=298), acute kidney injury within 3 months prior to ABPM (n=156), secondary hypertension (n=187), severe comorbidities (e.g., NYHA class IV heart failure, Child-Pugh class C cirrhosis, n=128), and missing key covariate data (n=100). The final study cohort included 2,143 patients, of whom 892 (41.6%) had biopsy-proven DKD and 1,251 (58.4%) had clinically diagnosed DKD (per 2022 KDIGO guidelines) after exclusion of other kidney diseases (3). Among the final cohort, 1,328 patients had serial ABPM measurements (median 2.7 measurements per patient over 2.1 years) and were included in the dynamic DBPV analysis.

DKD was diagnosed in accordance with the 2022 KDIGO guidelines, which define DKD as either biopsy-proven diabetic glomerulopathy or, in the absence of biopsy, the presence of type 1 diabetes (duration ≥10 years) or type 2 diabetes (duration ≥5 years) plus persistent albuminuria (urinary albumin-to-creatinine ratio [UACR] ≥30 mg/g) or eGFR <60 mL/min/1.73m^2^, after excluding other causes of kidney disease (3). Exclusion criteria for other kidney diseases included a history of glomerulonephritis (confirmed by serological markers or biopsy), hypertensive nephropathy (diagnosed in the absence of diabetes or with hypertension preceding diabetes by ≥5 years), renal amyloidosis, or polycystic kidney disease, as determined by a comprehensive clinical workup including serum and urine electrophoresis, autoimmune marker testing (e.g., antinuclear antibodies, anti-neutrophil cytoplasmic antibodies), and renal ultrasound.

Additional inclusion criteria were age ≥18 years, baseline eGFR 15–90 mL/min/1.73m^2^ (calculated using the CKD-EPI creatinine-cystatin C equation, which has been shown to improve accuracy in DKD compared to creatinine-only equations) (14), complete 24-hour ABPM data (defined as an effective recording rate ≥80%, with at least 20 valid daytime measurements and 7 valid nighttime measurements) (15), and availability of serial eGFR measurements (≥3 measurements over follow-up) to calculate annual decline rates. Exclusion criteria included secondary hypertension (e.g., primary aldosteronism, renal artery stenosis, Cushing’s syndrome), ESRD or renal replacement therapy (hemodialysis, peritoneal dialysis, or kidney transplantation) at baseline, acute kidney injury (AKI) within 3 months prior to ABPM (defined by KDIGO AKI criteria as an increase in serum creatinine ≥0.3 mg/dL within 48 hours or ≥1.5 times baseline within 7 days), severe heart failure (New York Heart Association class IV), liver cirrhosis (Child-Pugh class C), active malignancy (treatment within the past 2 years), pregnancy or lactation, and missing data on key covariates (e.g., HbA1c, UACR, lipid profile). To minimize residual confounding from unmeasured factors (e.g., sodium intake, medication adherence), we collected 24-hour urinary sodium excretion (available for 1,286 patients, 60.0%) and medication possession ratio (MPR, calculated as days of medication supply/days of follow-up, available for 1,952 patients, 91.1%) for sensitivity analyses, adjusting for these variables in additional models (16).

The study protocol was approved by the Institutional Review Board of the Seventh Affiliated Hospital of Sun Yat-sen University (IRB No. KY-2025-420-02) and was conducted in accordance with the Declaration of Helsinki. Informed consent was waived due to the retrospective nature of the study and the use of de-identified data, which were protected in accordance with institutional data security policies.

### 2.2 Data collection

Data were collected from electronic medical records (EMRs) using a standardized case report form (CRF) designed to capture demographic, clinical, laboratory, and outcome variables. Trained research personnel extracted data, which were then verified by a second investigator to ensure accuracy, with any discrepancies resolved through consensus with a senior clinician.

Baseline data were collected at the time of ABPM and included demographic characteristics (age, sex, body mass index [BMI] calculated as weight in kilograms divided by height in meters squared), smoking status (current smoker: ≥1 cigarette per day for the past 6 months; former smoker: quit ≥6 months prior; never smoker), and family history of DKD (first-degree relative with DKD). Clinical characteristics included diabetes type (type 1 or type 2), diabetes duration (self-reported or extracted from EMRs), hypertension duration (defined as a history of hypertension or use of antihypertensive medications), and history of cardiovascular disease (CVD), which included myocardial infarction, ischemic stroke, heart failure, or coronary artery bypass grafting, as confirmed by medical records or imaging studies.

Laboratory parameters were measured using standardized methods at clinical laboratory, which participated in external quality assurance programs to ensure consistency. Renal function parameters included serum creatinine (measured using an enzymatic method), serum cystatin C (measured using particle-enhanced immunoturbidimetry), eGFR (CKD-EPI creatinine-cystatin C equation), UACR (measured from a spot urine sample collected within 7 days of ABPM, using a turbidimetric immunoassay), and urinary β2-microglobulin (measured using a chemiluminescent immunoassay). Metabolic parameters included HbA1c (measured using high-performance liquid chromatography), fasting glucose (hexokinase method), total cholesterol, triglycerides, low-density lipoprotein cholesterol (LDL-C), high-density lipoprotein cholesterol (HDL-C) (all measured using enzymatic methods), and serum uric acid (urate oxidase method). Inflammatory markers included high-sensitivity C-reactive protein (hsCRP), measured using a nephelometric method, which was available for 1,689 (78.8%) patients. 24-hour urinary sodium excretion was measured using ion-selective electrode method (for patients with 24-hour urine collection within 7 days of ABPM), and MPR was extracted from pharmacy records to assess antihypertensive adherence (MPR ≥80% defined as good adherence).

Medication use was extracted from EMRs and included antihypertensive agents (RAAS inhibitors [angiotensin-converting enzyme inhibitors (ACEIs) or angiotensin II receptor blockers (ARBs)], calcium channel blockers [CCBs], beta-blockers, diuretics, mineralocorticoid receptor antagonists [MRAs]), antidiabetic agents (insulin, metformin, SGLT2 inhibitors, GLP-1 agonists, dipeptidyl peptidase-4 [DPP-4] inhibitors), statins, and antiplatelet agents (aspirin, clopidogrel). Medication use was defined as regular use (≥30 days prior to ABPM) and was documented as a binary variable (yes/no) for each class.

Follow-up data were collected through regular outpatient visits (scheduled every 3–6 months), telephone interviews (conducted annually for patients lost to outpatient follow-up), and linkage to the Guangdong Provincial Renal Replacement Therapy Registry—a comprehensive database that captures all incident ESRD cases and renal replacement therapy initiation in the province. Serial measurements of serum creatinine (every 3 months) and renal biomarkers (UACR, serum cystatin C, urinary β2-microglobulin, every 6 months) were extracted from EMRs to assess eGFR trends and biomarker changes. Serial ABPM was performed annually for patients with clinically indicated BP monitoring (e.g., uncontrolled BP, dose adjustments of antihypertensive medications), with a median of 2.7 measurements per patient over a median follow-up of 2.1 years. Adverse events, including ESRD, death, and CVD hospitalization, were documented with dates and causes verified through medical records, death certificates, or registry data, and patients were censored at the date of last follow-up, death from non-renal causes, or loss to follow-up.

### 2.3 Ambulatory blood pressure monitoring

ABPM was performed using two validated devices: the Omron HEM-7600CT (Omron Healthcare, Kyoto, Japan) and the Spacelabs 90217 (Spacelabs Healthcare, Redmond, WA, USA), both of which meet the International Protocol for Validation of Ambulatory Blood Pressure Monitors (15). Cuff size was selected based on arm circumference (small: 17–22 cm, medium: 22–32 cm, large: 32–42 cm) to ensure accurate measurements. Patients were instructed to wear the monitor continuously for 24 hours during their usual daily activities, avoiding strenuous exercise, alcohol consumption, and caffeine intake during the monitoring period, and they were also asked to maintain a sleep-wake diary, recording the times of going to bed and waking up, which were used to define daytime (06:00–22:00) and nighttime (22:00–06:00) periods.

ABPM measurements were taken every 30 minutes during the daytime and every 60 minutes during the nighttime, with a measurement considered valid if the systolic pressure was 70–260 mmHg and the diastolic pressure was 40–150 mmHg; measurements outside this range were excluded. The effective recording rate was calculated as the number of valid measurements divided by the total number of scheduled measurements, with a minimum effective recording rate of 80% required for inclusion.

DBPV parameters were calculated in accordance with the 2014 European Society of Hypertension (ESH) Practice Guidelines for Ambulatory Blood Pressure Monitoring and included 24-hour, daytime, and nighttime DBP SD (absolute variability, calculated as the standard deviation of all valid DBP measurements during the respective period in mmHg), 24-hour, daytime, and nighttime DBP CV (relative variability, calculated as [DBP SD/mean DBP] × 100% in %), 24-hour, daytime, and nighttime DBP ARV (a measure of short-term sequential variability calculated as the average of absolute differences between consecutive valid DBP measurements using the formula: $ARV=\frac{1}{\text{n-}1}\sum_{i=1}^{n-1}\left|DBP_{i+1}-DBP_{i}\right|$, where n is the number of valid DBP measurements during the respective period in mmHg), and nocturnal DBP decline rate (calculated as [(daytime mean DBP – nighttime mean DBP)/daytime mean DBP] × 100 in %) (17). Based on ESH guidelines, patients were categorized into three groups for nocturnal decline rate: non-dipping (decline <10%), dipping (decline 10–20%), and extreme dipping (decline >20%), with non-dipping treated as a binary variable (yes/no) and dipping and extreme dipping combined as the reference group for primary analysis. Systolic BPV parameters (24-hour, daytime, and nighttime SBP SD, CV, ARV) were also calculated for comparative analysis to assess the independent association of DBPV with outcomes after adjusting for SBPV.

Dynamic changes in DBPV were assessed in patients with ≥2 ABPM measurements (with an interval of ≥6 months between the first and last measurement) to ensure sufficient time to capture meaningful changes in BPV. For each patient, the annual change in 24-hour DBP SD was calculated as (final 24-hour DBP SD – baseline 24-hour DBP SD) divided by the follow-up duration (in years) between the first and last ABPM. Patients were categorized into three groups based on the annual change in 24-hour DBP SD, with thresholds aligned to prior studies in hypertension and chronic kidney disease that define clinically meaningful changes in BPV: decreased DBPV (annual change <–1 mmHg/year), stable DBPV (annual change between –1 and +1 mmHg/year), and increased DBPV (annual change >+1 mmHg/year). This categorization is supported by evidence that an annual change of ±1 mmHg/year in BPV correlates with significant differences in target organ damage risk (18), ensuring clinical relevance of the grouping. To verify the validity of dynamic DBPV grouping, we compared baseline 24-hour DBP SD between the three groups (decreased: 8.2 ± 1.9 mmHg; stable: 8.5 ± 2.1 mmHg; increased: 8.7 ± 2.2 mmHg, P = 0.12), confirming no baseline differences that could confound outcome associations.

### 2.4 Outcome definitions

The primary outcome was rapid eGFR decline, defined as an annual eGFR decline rate of ≥5 mL/min/1.73m^2^, with the annual eGFR decline rate calculated using linear regression of serial eGFR measurements (≥3 measurements) over the follow-up period and the slope of the regression line representing the annual decline rate. This threshold was chosen based on KDIGO guidelines, which identify an annual eGFR decline of ≥5 mL/min/1.73m^2^ as a marker of progressive CKD and a trigger for intensified clinical monitoring (3).

Secondary outcomes included ESRD (defined as an eGFR <15 mL/min/1.73m^2^ sustained for ≥3 months or initiation of renal replacement therapy [hemodialysis, peritoneal dialysis, or kidney transplantation], with the date of ESRD defined as the date of the first measurement of eGFR <15 mL/min/1.73m^2^ or the date of initiation of renal replacement therapy, whichever occurred first), composite renal events (defined as a ≥50% decline in eGFR from baseline sustained for ≥3 months or progression to ESRD, with a ≥50% eGFR decline chosen as a component of the composite outcome because it is a validated marker of severe renal function loss and a strong predictor of subsequent ESRD), and annual changes in renal biomarkers (annual changes in UACR [mg/g/year], serum cystatin C [mg/L/year], and urinary β2-microglobulin [mg/L/year], calculated using linear regression of serial biomarker measurements [≥3 measurements] over follow-up, with UACR and urinary β2-microglobulin log-transformed prior to analysis to normalize their distribution and results back-transformed for clinical interpretability).

### 2.5 Statistical analysis

All statistical analyses were performed using R software (version 4.3.2; R Foundation for Statistical Computing, Vienna, Austria) and SPSS 28.0 (IBM Corp., Armonk, NY, USA), with a two-sided *P*-value <0.05 considered statistically significant.

Continuous variables were assessed for normality using the Shapiro-Wilk test, with normally distributed variables presented as mean ± standard deviation (SD) and non-normally distributed variables (e.g., UACR, hsCRP, urinary β2-microglobulin) presented as median (interquartile range [IQR]). Categorical variables were presented as counts (percentages), and group comparisons were performed using one-way analysis of variance (ANOVA) for normally distributed continuous variables, the Kruskal-Wallis test for non-normally distributed continuous variables, and the chi-square test for categorical variables, with *post-hoc* pairwise comparisons performed using Tukey’s test for ANOVA and the Dunn-Bonferroni test for the Kruskal-Wallis test to adjust for multiple comparisons.

Multivariable logistic regression was used to assess the association between DBPV parameters and the odds of rapid eGFR decline, with three levels of adjustment: Model 1 adjusted for demographic factors (age, sex, BMI, smoking status); Model 2 adjusted for Model 1 plus metabolic and clinical factors (diabetes type, diabetes duration, hypertension duration, history of CVD, HbA1c, fasting glucose, LDL-C, HDL-C, triglycerides, serum uric acid, hsCRP [if available]); and Model 3 adjusted for Model 2 plus renal and hemodynamic factors (baseline eGFR, log-transformed UACR, 24-hour mean SBP, 24-hour mean DBP) and medication use (RAAS inhibitors, SGLT2 inhibitors, beta-blockers, statins).

Cox proportional hazards regression models were used to assess the association between DBPV parameters and time to ESRD or composite renal events, with the proportional hazards assumption verified using Schoenfeld residuals (through visual inspection of residual plots and formal testing using the Grambsch-Therneau test) and time-dependent covariates incorporated into the model if any parameter violated the assumption (none were identified in this study). The same three multivariable adjustment models used for logistic regression were applied to the Cox models.

Linear mixed-effects models were used to assess the association between DBPV parameters and continuous outcomes (annual eGFR decline rate and annual changes in renal biomarkers), with these models including random intercepts for individual patients to account for the correlation between repeated measurements within the same patient and fixed effects for DBPV parameters and covariates, and again using the same three covariate sets for adjustment.

The predictive performance of DBPV parameters was evaluated by comparing the performance of two models: a base model incorporating traditional risk factors (age, sex, diabetes type, diabetes duration, hypertension duration, history of CVD, HbA1c, LDL-C, baseline eGFR, log-transformed UACR, 24-hour mean SBP, RAAS inhibitor use, SGLT2 inhibitor use) and an augmented model adding DBPV parameters (24-hour DBP ARV, 24-hour DBP SD, or nocturnal non-dipping). Predictive performance was assessed using the C-statistic (a measure of discriminative ability with values ranging from 0.5 [no discrimination] to 1.0 [perfect discrimination], with differences tested using the DeLong test), net reclassification improvement (NRI, a measure of the improvement in reclassification of patients into risk categories [low: <5%, intermediate: 5–20%, high: >20%] based on 5-year ESRD risk), integrated discrimination improvement (IDI, a measure of the improvement in the mean difference between predicted probabilities for events and non-events), Akaike Information Criterion (AIC, a measure of model fit with lower values indicating better fit), time-dependent ROC curves (generated at 1-year, 3-year, and 5-year follow-up to assess discriminative ability over time, with AUC calculated for each time point and differences tested using the DeLong test), and decision curve analysis (DCA, used to assess clinical net benefit by comparing the expected benefit of treating patients based on the model’s predictions versus treating all patients or no patients, with net benefit calculated as [true positives – false positives × threshold probability/(1 – threshold probability)] divided by the total number of patients and threshold probabilities ranging from 0 to 1).

Effect modification was assessed by including interaction terms between DBPV parameters (24-hour DBP ARV, chosen as the most predictive parameter) and potential effect modifiers (DKD stage: eGFR ≥60 vs. <60 mL/min/1.73m^2^; albuminuria strata: moderately increased albuminuria vs. severely increased albuminuria; diabetes type: type 1 vs. type 2; RAAS inhibitor use: yes vs. no) in the Cox proportional hazards models for ESRD, with the statistical significance of interaction terms tested using likelihood ratio tests (comparing models with and without the interaction term) and stratified analyses performed for significant effect modifiers to quantify the association between DBPV and ESRD in each stratum. Additionally, we tested the interaction between 24-hour DBP ARV and 24-hour SBP ARV to explore whether their combined effect amplified ESRD risk. We also performed ROC curve analysis (with Youden index) to determine the optimal threshold of 24-hour DBP ARV for ESRD prediction, and subgroup analysis to compare DBPV parameters across different antihypertensive medication classes (RAAS inhibitors, CCBs, beta-blockers, diuretics).

Several sensitivity analyses were performed to assess the robustness of findings: exclusion of patients with baseline AKI history (n=156) to ensure baseline renal function was stable; exclusion of beta-blocker users (n=428) to assess whether beta-blocker use confounded the association between DBPV and outcomes; use of an alternative definition of rapid eGFR decline (≥7.5 mL/min/1.73m^2^/year) to confirm consistency across thresholds; adjustment for 24-hour SBP ARV to assess the independent association of DBPV; restriction to biopsy-proven DKD (n=892) to avoid misclassification; adjustment for 24-hour urinary sodium excretion and MPR to control for unmeasured confounding; and internal validation using 1,000 Bootstrap samples to assess for overfitting (with C-statistic, NRI, and IDI calculated for each sample and median and 95% CI reported).

## 3 Results

### 3.1 Baseline characteristics of the study population

A total of 2,143 patients with DKD were included in the study, with a mean age of 57.2 ± 11.5 years and 63.1% male. The majority of patients (84.1%) had type 2 diabetes, with a median diabetes duration of 10.8 years (IQR 6.8–16.5 years), and hypertension was highly prevalent (89.2% of patients), with a mean hypertension duration of 7.5 ± 4.2 years and 23.5% of patients having a history of cardiovascular disease. The mean baseline eGFR was 52.9 ± 18.6 mL/min/1.73m^2^, and the median UACR was 336 mg/g (IQR 105–892 mg/g), indicating that most patients had moderate to advanced DKD.

**Table 1** compares baseline characteristics between patients with serial ABPM and single ABPM: no significant differences were observed in core prognostic factors, including age (57.0 ± 11.6 vs. 57.5 ± 11.4 years, *P* = 0.42), eGFR (53.2 ± 18.5 vs. 52.5 ± 18.7 mL/min/1.73m^2^, *P* = 0.51), UACR (342 ± 286 vs. 331 ± 278 mg/g, *P* = 0.38), and 24-hour DBP ARV (9.8 ± 2.4 vs. 10.1 ± 2.6 mmHg, *P* = 0.11). Among medication use, the prevalence of RAAS inhibitors, CCBs, SGLT2 inhibitors, and statins was comparable between groups (all *P*>0.05); only beta-blocker use showed a small statistically significant difference (26.9% vs. 22.3%, *P* = 0.02), but the absolute difference was minimal (4.6%) and this variable was adjusted for in all subsequent multivariable models. Collectively, these results rule out significant selection bias in the dynamic DBPV analysis.

**Table 1:** Baseline characteristics of patients with serial vs. single ABPM.

Characteristic	Serial ABPM (n=1,328)	Single ABPM (n=815)	*P*-value
Age, years	57.0 ± 11.6	57.5 ± 11.4	0.42
Sex (male), n (%)	838 (63.1%)	501 (61.5%)	0.53
BMI, kg/m^2^	25.8 ± 3.4	25.6 ± 3.3	0.31
eGFR, mL/min/1.73m^2^	53.2 ± 18.5	52.5 ± 18.7	0.51
UACR, mg/g	342 ± 286	331 ± 278	0.38
24-hour mean SBP, mmHg	138.7 ± 15.2	139.1 ± 15.4	0.67
24-hour mean DBP, mmHg	78.9 ± 9.2	79.2 ± 9.3	0.58
24-hour DBP ARV, mmHg	9.8 ± 2.4	10.1 ± 2.6	0.11
Nocturnal non-dipping, n (%)	721 (54.3%)	443 (54.4%)	0.98
Medication Use			
RAAS inhibitor use, n (%)	892 (67.2%)	514 (63.1%)	0.08
CCB use, n (%)	586 (44.1%)	382 (46.9%)	0.21
Beta-blocker use, n (%)	358 (26.9%)	182 (22.3%)	0.02
SGLT2 inhibitor use, n (%)	165 (12.4%)	98 (12.0%)	0.85
Statin use, n (%)	826 (62.2%)	501 (61.5%)	0.79

Data are presented as mean ± SD or n (%). *P*-values calculated via t-test (continuous variables) or chi-square test (categorical variables).

**Table 2** presents the baseline characteristics of the study population stratified by 24-hour DBP ARV tertiles, as 24-hour DBP ARV emerged as the most predictive DBPV parameter in subsequent analyses. Patients in the highest 24-hour DBP ARV tertile (tertile 3: >10.5 mmHg) were older, with a mean age of 59.8 ± 11.8 years compared to 54.6 ± 10.9 years in the lowest tertile, and they had longer diabetes duration, with a median of 12.1 years (IQR 7.8–17.5) versus 9.2 years (IQR 5.5–14.1) in the lowest tertile. Glycemic control was also worse in the highest tertile, with a mean HbA1c of 8.9 ± 1.9% compared to 7.8 ± 1.5% in the lowest tertile.

**Table 2:** Baseline characteristics of the study population stratified by 24-hour DBP ARV tertiles.

Characteristic	Tertile 1 (n=714)	Tertile 2 (n=715)	Tertile 3 (n=714)	*P*-value
Demographics				
Age, years	54.6 ± 10.9	57.3 ± 11.4	59.8 ± 11.8	<0.001
Male sex, n (%)	428 (59.9)	451 (63.1)	480 (67.2)	0.004
BMI, kg/m^2^	25.1 ± 3.2	25.8 ± 3.4	26.5 ± 3.6	<0.001
Smoking status, n (%) 0.012				
Never smoker	412 (57.7)	398 (55.7)	376 (52.7)	
Former smoker	184 (25.8)	192 (26.9)	195 (27.3)	
Current smoker	118 (16.5)	125 (17.5)	143 (20.0)	
Clinical History				
Diabetes type, n (%)				0.103
Type 1	112 (15.7)	105 (14.7)	98 (13.7)	
Type 2	602 (84.3)	610 (85.3)	616 (86.3)	
Diabetes duration, years	9.2 [5.5–14.1]	10.9 [6.8–15.7]	12.1 [7.8–17.5]	<0.001
Hypertension duration, years	6.8 ± 3.9	7.5 ± 4.1	8.2 ± 4.4	<0.001
History of CVD, n (%)	148 (20.7)	165 (23.1)	198 (27.7)	0.002
Laboratory Parameters				
HbA1c, %	7.8 ± 1.5	8.4 ± 1.7	8.9 ± 1.9	<0.001
Fasting glucose, mmol/L	7.6 ± 1.8	8.2 ± 2.1	8.8 ± 2.3	<0.001
LDL-C, mmol/L	2.6 ± 0.7	2.8 ± 0.8	3.0 ± 0.9	<0.001
HDL-C, mmol/L	1.3 ± 0.3	1.2 ± 0.3	1.1 ± 0.3	<0.001
Serum uric acid, μmol/L	392 ± 84	421 ± 89	458 ± 95	<0.001
hsCRP, mg/L^2^	2.8 [1.6–4.9]	3.2 [1.8–5.6]	3.8 [2.1–6.5]	<0.001
eGFR, mL/min/1.73m^2^	60.5 ± 18.3	52.7 ± 17.8	47.2 ± 17.9	<0.001
UACR, mg/g	208 [72–564]	295 [118–782]	452 [156–1138]	<0.001
Serum cystatin C, mg/L	1.3 ± 0.4	1.5 ± 0.5	1.8 ± 0.6	<0.001
Urinary β2-microglobulin, mg/L	0.5 [0.3–0.8]	0.7 [0.4–1.0]	0.9 [0.6–1.3]	<0.001
ABPM Metrics				
24-hour mean SBP, mmHg	133.8 ± 13.9	138.6 ± 15.1	144.3 ± 16.2	<0.001
24-hour mean DBP, mmHg	75.2 ± 8.3	78.9 ± 9.2	82.8 ± 10.1	<0.001
Nocturnal DBP decline rate, %	12.8 ± 4.5	9.7 ± 3.8	6.9 ± 3.2	<0.001
Nocturnal non-dipping, n (%)	213 (29.8)	435 (60.8)	516 (72.3)	<0.001
Medication Use				
RAAS inhibitors	597 (83.5)	532 (74.4)	479 (67.2)	<0.001
SGLT2 inhibitors	165 (23.1)	158 (22.1)	156 (21.8)	0.826
β-blockers	120 (16.8)	189 (26.4)	232 (32.5)	<0.001
Statins	425 (59.5)	431 (60.3)	438 (61.3)	0.765

Tertile 1, ≤7.8 mmHg; Tertile 2, 7.9–10.5 mmHg; Tertile 3, >10.5 mmHg. Data are presented as mean ± SD, median [interquartile range (IQR)], or n (%). *P*-values were calculated using one-way ANOVA (normally distributed continuous variables), Kruskal-Wallis test (non-normally distributed continuous variables), or chi-square test (categorical variables). BMI, body mass index; CVD, cardiovascular disease; HbA1c, glycated hemoglobin; LDL-C, low-density lipoprotein cholesterol; HDL-C, high-density lipoprotein cholesterol; hsCRP, high-sensitivity C-reactive protein; eGFR, estimated glomerular filtration rate; UACR, urinary albumin-to-creatinine ratio; DBP, diastolic blood pressure; ARV, average real variability; RAAS, renin-angiotensin-aldosterone system; SGLT2, sodium-glucose cotransporter-2. ^2^hsCRP data available for 1,689 patients (562 in Tertile 1, 558 in Tertile 2, 569 in Tertile 3).

Renal function differed significantly across tertiles, with the highest ARV tertile having a lower mean eGFR (47.2 ± 17.9 mL/min/1.73m^2^ vs. 60.5 ± 18.3 mL/min/1.73m^2^ in the lowest tertile), a higher median UACR (452 mg/g [IQR 156–1,138] vs. 208 mg/g [IQR 72–564] in the lowest tertile), and a higher serum cystatin C (1.8 ± 0.6 mg/L vs. 1.3 ± 0.4 mg/L in the lowest tertile). Hemodynamically, patients in the highest ARV tertile had higher 24-hour mean SBP (144.3 ± 16.2 mmHg vs. 133.8 ± 13.9 mmHg in the lowest tertile) and 24-hour mean DBP (82.8 ± 10.1 mmHg vs. 75.2 ± 8.3 mmHg in the lowest tertile), as well as a higher prevalence of nocturnal non-dipping (72.3% vs. 29.8% in the lowest tertile).

Medication use also varied across tertiles: RAAS inhibitor use was lower in the highest ARV tertile (67.2% vs. 83.5% in the lowest tertile), while beta-blocker use was higher (32.5% vs. 16.8% in the lowest tertile). Statin use was similar across tertiles (approximately 60%), as was SGLT2 inhibitor use (approximately 23%). Inflammatory markers aligned with DBPV, with the highest ARV tertile having a higher median hsCRP (3.8 mg/L [IQR 2.1–6.5] vs. 2.8 mg/L [IQR 1.6–4.9] in the lowest tertile), suggesting a potential link between inflammation and BPV in DKD.

### 3.2 Association between baseline DBPV and rapid eGFR decline

Over a median follow-up of 4.8 years, 896 (41.8%) patients experienced rapid eGFR decline (annual decline ≥5 mL/min/1.73m^2^). The median annual eGFR decline rate varied significantly across 24-hour DBP ARV tertiles: 1.7 mL/min/1.73m^2^ (IQR 0.6–3.9) in tertile 1, 3.2 mL/min/1.73m^2^ (IQR 1.3–5.8) in tertile 2, and 6.1 mL/min/1.73m^2^ (IQR 3.1–9.2) in tertile 3 (*P* < 0.001 for overall comparison).

**Table 3** presents the associations between all DBPV parameters and rapid eGFR decline, as assessed by multivariable logistic regression. After full adjustment in Model 3—accounting for demographic, metabolic, renal, hemodynamic, and medication factors—24-hour DBP ARV was the strongest predictor of rapid eGFR decline, with each 1 mmHg increase associated with an 18% higher odds of rapid decline (OR 1.18, 95% CI 1.13–1.23, *P* < 0.001). Other DBPV parameters also predicted rapid decline, including 24-hour DBP SD (OR 1.12, 95% CI 1.07–1.17, *P* < 0.001), 24-hour DBP CV (OR 1.09, 95% CI 1.06–1.12, *P* < 0.001), and nocturnal non-dipping (OR 1.58, 95% CI 1.32–1.89, *P* < 0.001).

**Table 3:** Associations between DBPV parameters and rapid eGFR decline (annual decline ≥5 mL/min/1.73m^2^).

DBPV Parameter	Model 1	Model 2	Model 3
24-hour DBP			
SD (per 1 mmHg increase)	OR=1.16 (1.12–1.20), *P* < 0.001	OR=1.14 (1.10–1.18), *P* < 0.001	OR=1.12 (1.07–1.17), *P* < 0.001
CV (per 1% increase)	OR=1.12 (1.09–1.15), *P* < 0.001	OR=1.11 (1.08–1.14), *P* < 0.001	OR=1.09 (1.06–1.12), *P* < 0.001
ARV (per 1 mmHg increase)	OR=1.22 (1.17–1.27), *P* < 0.001	OR=1.20 (1.15–1.25), *P* < 0.001	OR=1.18 (1.13–1.23), *P* < 0.001
Daytime DBP			
SD (per 1 mmHg increase)	OR=1.14 (1.10–1.18), *P* < 0.001	OR=1.12 (1.08–1.16), *P* < 0.001	OR=1.10 (1.05–1.14), *P* < 0.001
CV (per 1% increase)	OR=1.10 (1.07–1.13), *P* < 0.001	OR=1.09 (1.06–1.12), *P* < 0.001	OR=1.08 (1.05–1.11), *P* < 0.001
ARV (per 1 mmHg increase)	OR=1.18 (1.13–1.23), *P* < 0.001	OR=1.15 (1.10–1.20), *P* < 0.001	OR=1.10 (1.05–1.15), *P* < 0.001
SD (per 1 mmHg increase)	OR=1.18 (1.13–1.23), *P* < 0.001	OR=1.16 (1.11–1.21), *P* < 0.001	OR=1.14 (1.09–1.19), *P* < 0.001
Nighttime DBP			
CV (per 1% increase)	OR=1.14 (1.11–1.17), *P* < 0.001	OR=1.12 (1.09–1.15), *P* < 0.001	OR=1.10 (1.07–1.13), *P* < 0.001
ARV (per 1 mmHg increase)	OR=1.24 (1.19–1.29), *P* < 0.001	OR=1.21 (1.16–1.26), *P* < 0.001	OR=1.16 (1.11–1.21), *P* < 0.001
Nocturnal non-dipping (ref: dipping/extreme dipping)	OR=1.85 (1.57–2.18), *P* < 0.001	OR=1.72 (1.45–2.04), *P* < 0.001	OR=1.58 (1.32–1.89), *P* < 0.001

Data are presented as odds ratio (OR) and 95% confidence interval (CI). Rapid eGFR decline occurred in 896 (41.8%) patients. Model 1, Adjusted for age, sex, BMI, and smoking status. Model 2, Adjusted for Model 1 + diabetes type, diabetes duration, hypertension duration, history of CVD, HbA1c, fasting glucose, LDL-C, HDL-C, triglycerides, serum uric acid, and hsCRP (if available). Model 3, Adjusted for Model 2 + baseline eGFR, log-transformed UACR, 24-hour mean SBP, 24-hour mean DBP, RAAS inhibitor use, SGLT2 inhibitor use, β-blocker use, and statin use. DBPV, diastolic blood pressure variability; SD, standard deviation; CV, coefficient of variation; ARV, average real variability; ref, reference.

Notably, nighttime DBPV parameters were more strongly associated with rapid eGFR decline than daytime parameters. For example, nighttime DBP ARV was associated with an OR of 1.16 (95% CI 1.11–1.21, *P* < 0.001) per 1 mmHg increase, compared to 1.10 (95% CI 1.05–1.15, *P* < 0.001) for daytime DBP ARV (P<0.05 for comparison of ORs), a finding that underscores the importance of nocturnal BPV in DKD progression and aligns with the circadian regulation of renal function.

### 3.3 Association between baseline DBPV and secondary renal outcomes

During follow-up, 352 (16.4%) patients progressed to ESRD, and 573 (26.7%) developed composite renal events (≥50% eGFR decline or ESRD). The cumulative incidence of ESRD at 5 years varied significantly across 24-hour DBP ARV tertiles: 8.2% (95% CI 5.9–10.5%) in tertile 1, 16.5% (95% CI 13.4–19.6%) in tertile 2, and 28.7% (95% CI 24.9–32.5%) in tertile 3 (log-rank *P* < 0.001).

**Figure 2:**
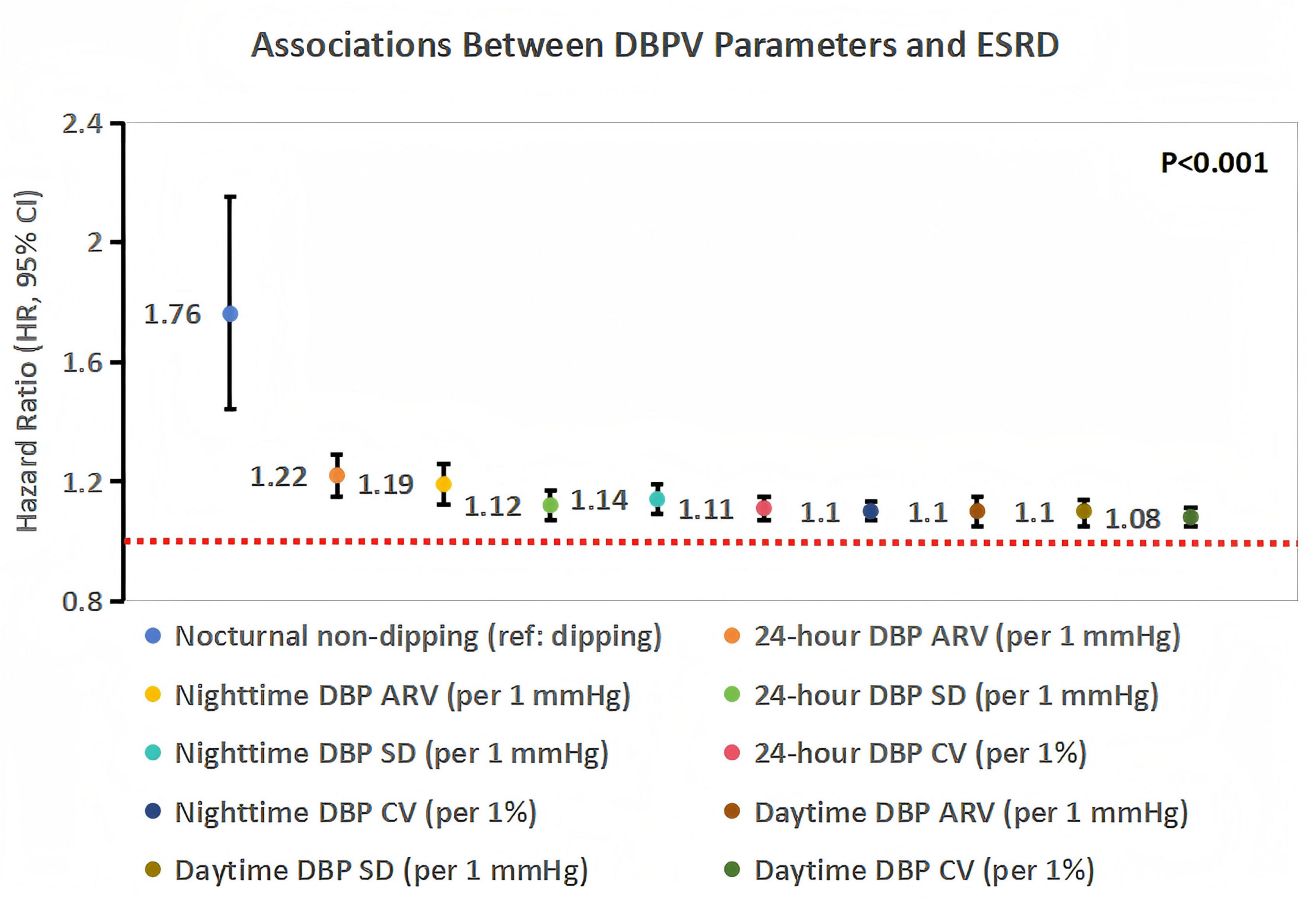
Forest plot of associations between diastolic blood pressure variability (DBPV) parameters and end-stage renal disease (ESRD) in diabetic kidney disease (DKD) patients. Analyses were performed using multivariable Cox regression (adjusted for demographics, metabolic factors, renal function, and medications). Squares represent hazard ratios (HRs; size proportional to statistical weight), horizontal lines denote 95% confidence intervals (CIs). All DBPV parameters were significantly associated with ESRD (P<0.001).

**Figure 3:**
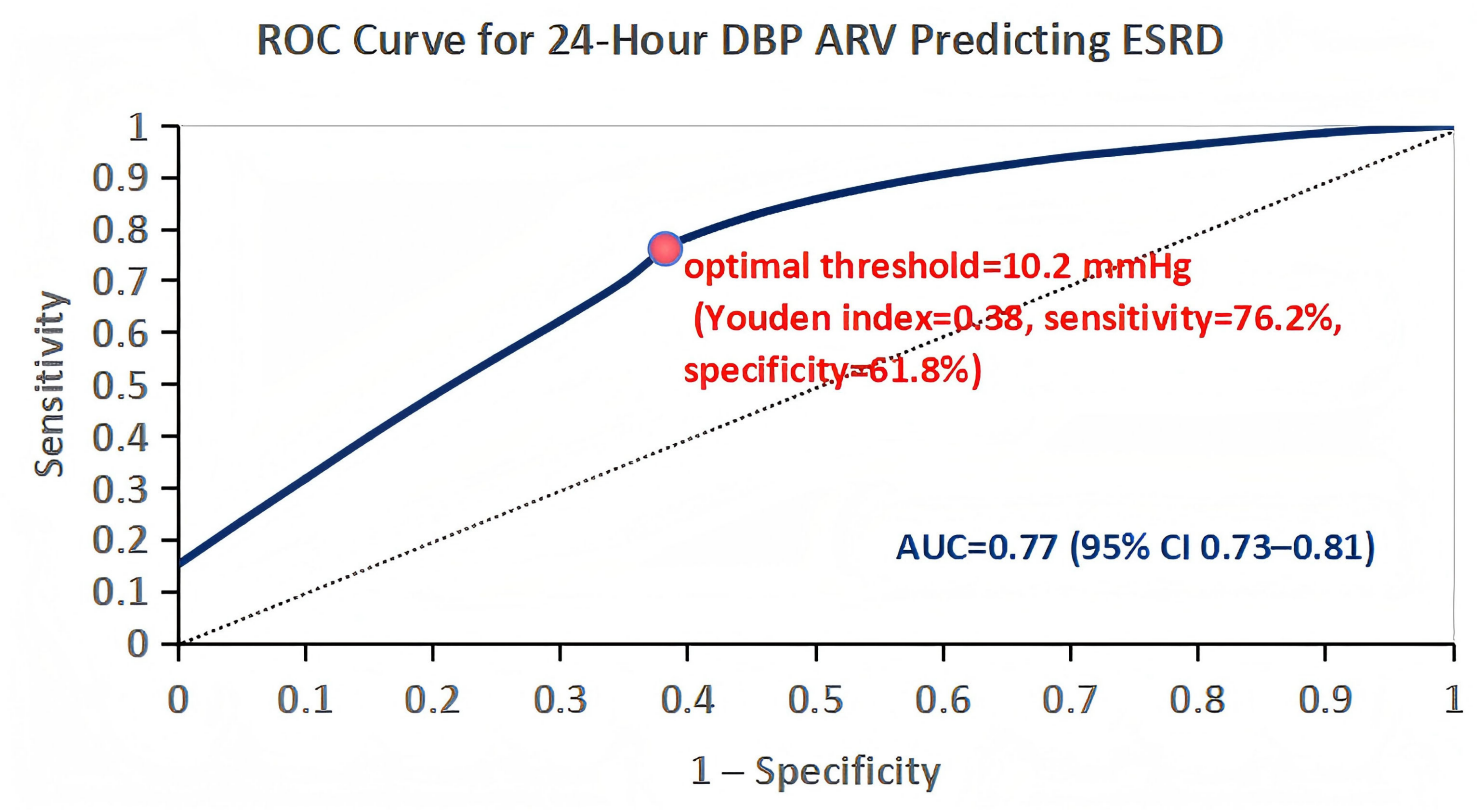
ROC curve for predicting end-stage renal disease (ESRD) using 24-hour diastolic blood pressure average real variability (DBP ARV) in patients with diabetic kidney disease (DKD). The area under the curve (AUC) was 0.77 (95% confidence interval: 0.73–0.81), with the optimal threshold of 24-hour DBP ARV identified as 10.2 mmHg (Youden index = 0.38) corresponding to a sensitivity of 76.2% and specificity of 61.8%.

**Figure 2** presents the forest plot of associations between DBPV parameters and ESRD, as assessed by multivariable Cox regression (Model 3). 24-hour DBP ARV was the most strongly associated parameter, with each 1 mmHg increase in 24-hour DBP ARV linked to a 22% higher risk of ESRD (HR 1.22, 95% CI 1.15–1.29, *P* < 0.001). **Figure 3** shows the ROC curve for 24-hour DBP ARV predicting ESRD: the area under the curve (AUC) was 0.77 (95% CI 0.73–0.81), and the internally derived hypothesis-generating 24-hour DBP ARV threshold was 10.2 mmHg (Youden index=0.38, sensitivity=76.2%, specificity=61.8%). Patients with 24-hour DBP ARV >10.2 mmHg had a 3.1-fold higher risk of ESRD than those with ≤10.2 mmHg (HR 3.10, 95% CI 2.52–3.82, *P* < 0.001) after full adjustment. Nocturnal non-dipping was also a strong predictor, with an HR of 1.76 (95% CI 1.44–2.15, *P* < 0.001) compared to dipping or extreme dipping, while other DBPV parameters—including 24-hour DBP SD (HR 1.12, 95% CI 1.07–1.17, *P* < 0.001) and 24-hour DBP CV (HR 1.11, 95% CI 1.07–1.15, *P* < 0.001)—were also independently associated with ESRD.

When adjusting for 24-hour SBP ARV to isolate the independent contribution of DBPV, the association between 24-hour DBP ARV and ESRD remained significant (HR 1.19, 95% CI 1.12–1.26, *P* < 0.001), while 24-hour SBP ARV was associated with a smaller increase in risk (HR 1.07, 95% CI 1.01–1.13, *P* = 0.02). This finding confirms that DBPV is an independent predictor of ESRD, beyond the effects of SBPV. The interaction between 24-hour DBP ARV and 24-hour SBP ARV on ESRD risk was not significant (*P*-interaction=0.23), but patients with both 24-hour DBP ARV >10.2 mmHg and 24-hour SBP ARV >12.5 mmHg (optimal threshold for SBP ARV, derived from ROC) had a 4.5-fold higher ESRD risk than those with both parameters below thresholds (HR 4.50, 95% CI 3.61–5.62, *P* < 0.001), indicating additive risk of combined high DBPV and SBPV.

The associations between DBPV parameters and composite renal events were similar to those observed for ESRD. 24-hour DBP ARV was associated with an HR of 1.20 (95% CI 1.14–1.26, *P* < 0.001) per 1 mmHg increase, while nocturnal non-dipping was associated with an HR of 1.51 (95% CI 1.26–1.81, *P* < 0.001), further supporting the link between DBPV and severe renal function loss.

The **Table 4** presents the associations between 24-hour DBP ARV and annual changes in renal biomarkers, as assessed by linear mixed-effects models (Model 3). Higher 24-hour DBP ARV was associated with progressive worsening of all renal biomarkers: each 1 mmHg increase in 24-hour DBP ARV was linked to an annual increase in UACR of 8.2 mg/g (β = 8.2, 95% CI 5.7–10.7, *P* < 0.001)—a change that translates to a 12% annual increase in UACR for a 5 mmHg increase in ARV, a clinically meaningful trend given UACR’s role as a predictor of ESRD. Additionally, each 1 mmHg increase in 24-hour DBP ARV was associated with an annual increase in serum cystatin C of 0.06 mg/L (β = 0.06, 95% CI 0.04–0.08, *P* < 0.001)—a marker of early renal decline that is more sensitive than serum creatinine in DKD—and an annual increase in urinary β2-microglobulin of 0.05 mg/L (β = 0.05, 95% CI 0.03–0.07, *P* < 0.001), which reflects tubular injury and confirms that DBPV impacts both glomerular and tubular function in DKD. These associations remained significant after adjusting for 24-hour SBP ARV, reinforcing the independent role of DBPV in biomarker progression.

**Table 4:** Associations between 24-hour DBP ARV and annual changes in renal biomarkers.

Renal biomarker	Annual change (β, 95% CI)	*P*-value
UACR, mg/g/year	8.2 (5.7–10.7)	<0.001
Serum cystatin C, mg/L/year	0.06 (0.04–0.08)	<0.001
Urinary β2-microglobulin, mg/L/year	0.05 (0.03–0.07)	<0.001

Data are presented as β-coefficient and 95% CI. Analyses were performed using linear mixed-effects models adjusted for Model 3 covariates (age, sex, BMI, smoking status, diabetes type/duration, hypertension duration, history of CVD, HbA1c, fasting glucose, LDL-C, HDL-C, triglycerides, serum uric acid, hsCRP, baseline eGFR, log-transformed UACR, 24-hour mean SBP/DBP, and medication use). Random intercepts were included to account for repeated measurements within patients. DBP ARV, diastolic blood pressure average real variability; UACR, urinary albumin-to-creatinine ratio.

**Table 5** presents DBPV parameters across antihypertensive medication classes: patients using CCBs (n=586) had lower 24-hour DBP ARV (8.9 ± 2.3 mmHg) than those using RAAS inhibitors alone (n=724, 10.1 ± 2.5 mmHg, *P* = 0.003) or beta-blockers alone (n=218, 11.4 ± 2.8 mmHg, *P* < 0.001). No significant difference was observed between RAAS inhibitors + CCBs (n=342, 9.2 ± 2.4 mmHg) and CCBs alone (*P* = 0.28).

**Table 5:** 24-hour DBP ARV across antihypertensive medication classes.

Medication class	n	24-hour DBP ARV (mmHg, mean ± SD)	*P*-value vs. CCBs
CCBs alone	586	8.9 ± 2.3	–
RAAS inhibitors alone	724	10.1 ± 2.5	0.003
Beta-blockers alone	218	11.4 ± 2.8	<0.001
Diuretics alone	152	10.8 ± 2.6	<0.001
RAAS inhibitors + CCBs	342	9.2 ± 2.4	0.28
RAAS inhibitors + beta-blockers	286	10.5 ± 2.7	0.001

CCBs, calcium channel blockers; RAAS inhibitors, angiotensin-converting enzyme inhibitors or angiotensin II receptor blockers. *P*-values calculated via one-way ANOVA with Tukey’s *post-hoc* test.

### 3.4 Association between dynamic changes in DBPV and renal outcomes

Among 1,328 patients with serial ABPM measurements (median 2.7 measurements over 2.1 years), 412 (31.0%) had increased DBPV (annual change in 24-hour DBP SD >+1 mmHg/year), 453 (34.1%) had stable DBPV (annual change between –1 and +1 mmHg/year), and 463 (34.9%) had decreased DBPV (annual change <–1 mmHg/year). To verify the validity of dynamic DBPV grouping, we compared baseline 24-hour DBP SD between the three groups (decreased: 8.2 ± 1.9 mmHg; stable: 8.5 ± 2.1 mmHg; increased: 8.7 ± 2.2 mmHg, *P* = 0.12), confirming no baseline differences that could confound outcome associations.

**Figure 4:**
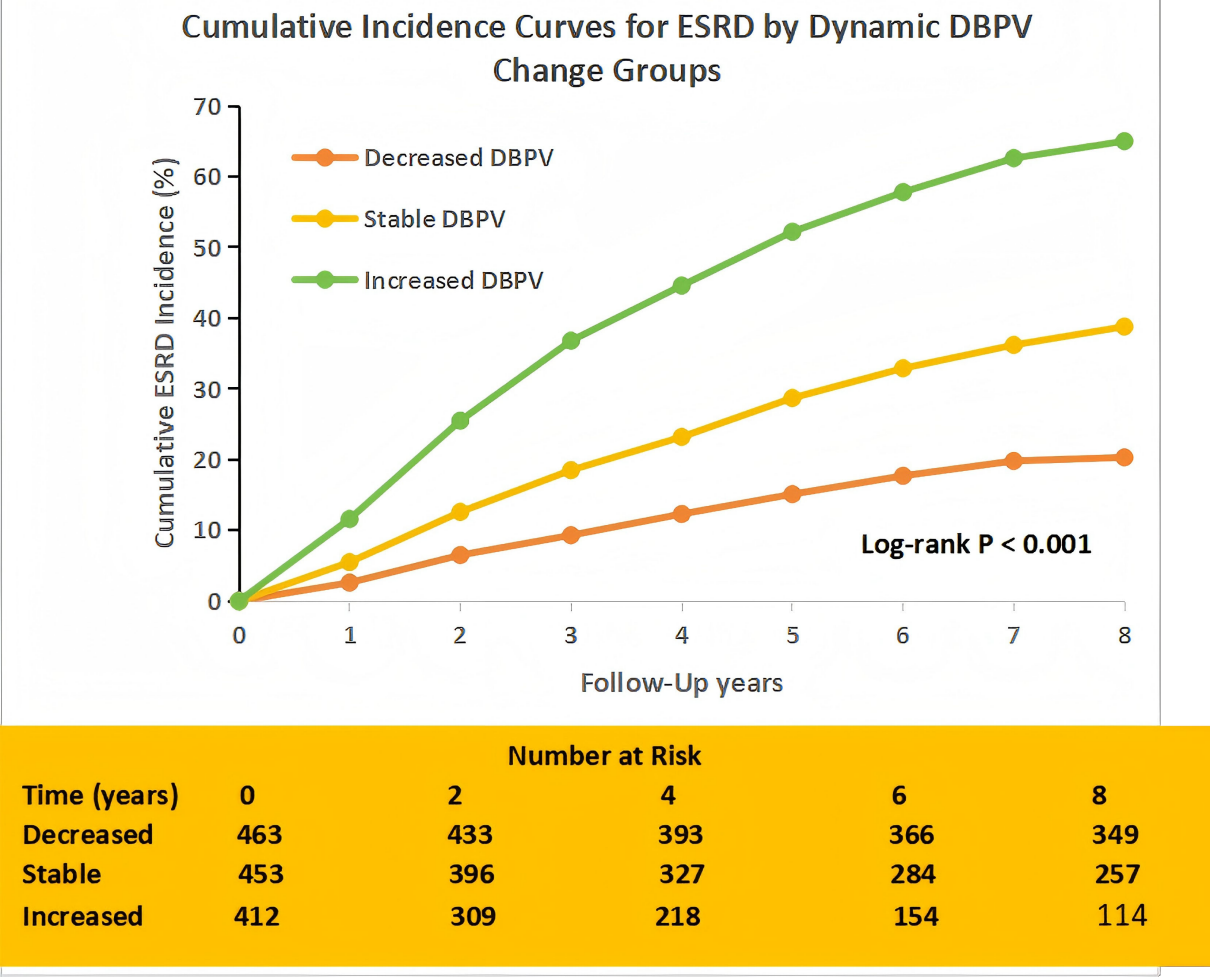
ESRD cumulative incidence by dynamic DBPV change groups (Decreased: annual 24-hour DBP SD <–1 mmHg/year; Stable: –1 to +1 mmHg/year; Increased: >1 mmHg/year). Follow-up was truncated at 8 years. Numbers at risk are shown below the x-axis. Log-rank test for intergroup difference: P<0.001.

The cumulative incidence of ESRD differed significantly across DBPV change groups: 9.5% (95% CI 6.8–12.2%) in the decreased DBPV group, 17.3% (95% CI 13.9–20.7%) in the stable group, and 32.1% (95% CI 27.8–36.4%) in the increased group (log-rank *P* < 0.001; **Figure 4**). After multivariable adjustment (Model 3), increased DBPV was associated with a 2.4-fold higher risk of ESRD compared to decreased DBPV (HR 2.40, 95% CI 1.92–3.00, *P* < 0.001), while stable DBPV was associated with a 1.45-fold higher risk (HR 1.45, 95% CI 1.15–1.83, *P* = 0.002).

Similar associations were observed for composite renal events: the cumulative incidence was 15.1% (95% CI 11.9–18.3%) in the decreased group, 22.8% (95% CI 19.1–26.5%) in the stable group, and 39.4% (95% CI 34.8–44.0%) in the increased group (log-rank *P* < 0.001), with increased DBPV linked to an HR of 1.82 (95% CI 1.50–2.21, *P* < 0.001) for composite events compared to decreased DBPV.

The annual eGFR decline rate also varied across DBPV change groups: 1.9 ± 1.8 mL/min/1.73m^2^ in the decreased group, 3.5 ± 2.1 mL/min/1.73m^2^ in the stable group, and 6.8 ± 2.7 mL/min/1.73m^2^ in the increased group (P<0.001 for overall comparison). After multivariable adjustment, increased DBPV was associated with a significantly faster annual eGFR decline compared to decreased DBPV (β = –2.9 mL/min/1.73m^2^, 95% CI –3.5 to –2.3, *P* < 0.001), while stable DBPV was associated with a smaller decline (β = –1.6 mL/min/1.73m^2^, 95% CI –2.2 to –1.0, *P* < 0.001). These findings highlight the clinical importance of dynamic changes in DBPV, as even modest increases in DBPV over time are associated with a substantially higher risk of adverse renal outcomes.

### 3.5 Predictive performance of DBPV parameters

**Table 6** presents the predictive performance of models for ESRD, comparing the base model (traditional risk factors) with augmented models adding DBPV parameters. Adding 24-hour DBP ARV to the base model significantly improved discriminative ability, with the C-statistic increasing from 0.73 (95% CI 0.69–0.77) to 0.80 (95% CI 0.76–0.84, *P* < 0.001). The NRI was 0.28 (95% CI 0.21–0.35, *P* < 0.001), indicating that 28% of patients were correctly reclassified into higher or lower risk categories when 24-hour DBP ARV was added, and the IDI was 0.05 (95% CI 0.03–0.07, *P* < 0.001), reflecting an improvement in the mean difference between predicted probabilities for events and non-events. The AIC also decreased from 2,845 to 2,789, indicating better model fit.

**Table 6:** Predictive performance of models for ESRD.

Model	C-statistic (95% CI)	NRI (95% CI)	IDI (95% CI)	AIC	*P*-value
Base model	0.73 (0.69–0.77)	—	—	2845	—
Base + 24-hour DBP ARV	0.80 (0.76–0.84)	0.28 (0.21–0.35)	0.05 (0.03–0.07)	2789	<0.001
Base + 24-hour DBP SD	0.77 (0.73–0.81)	0.21 (0.14–0.28)	0.03 (0.02–0.05)	2816	<0.001
Base + Nocturnal non-dipping	0.75 (0.71–0.79)	0.18 (0.11–0.25)	0.02 (0.01–0.04)	2832	<0.001

ESRD occurred in 352 (16.4%) patients. The base model included age, sex, diabetes type, diabetes duration, hypertension duration, history of CVD, HbA1c, LDL-C, baseline eGFR, log-transformed UACR, 24-hour mean SBP, RAAS inhibitor use, and SGLT2 inhibitor use. P-values compare the augmented model to the base model. ESRD, end-stage renal disease; NRI, net reclassification improvement; IDI, integrated discrimination improvement; AIC, Akaike Information Criterion; DBP, diastolic blood pressure; ARV, average real variability; SD, standard deviation.

Adding other DBPV parameters—including 24-hour DBP SD and nocturnal non-dipping—also improved predictive performance, but to a lesser extent than 24-hour DBP ARV. For example, adding 24-hour DBP SD increased the C-statistic to 0.77 (95% CI 0.73–0.81, *P* < 0.001), with an NRI of 0.21 (95% CI 0.14–0.28, *P* < 0.001) and IDI of 0.03 (95% CI 0.02–0.05, *P* < 0.001), while adding nocturnal non-dipping increased the C-statistic to 0.75 (95% CI 0.71–0.79, *P* < 0.001), with an NRI of 0.18 (95% CI 0.11–0.25, *P* < 0.001) and IDI of 0.02 (95% CI 0.01–0.04, *P* < 0.001).

**Figure 5:**
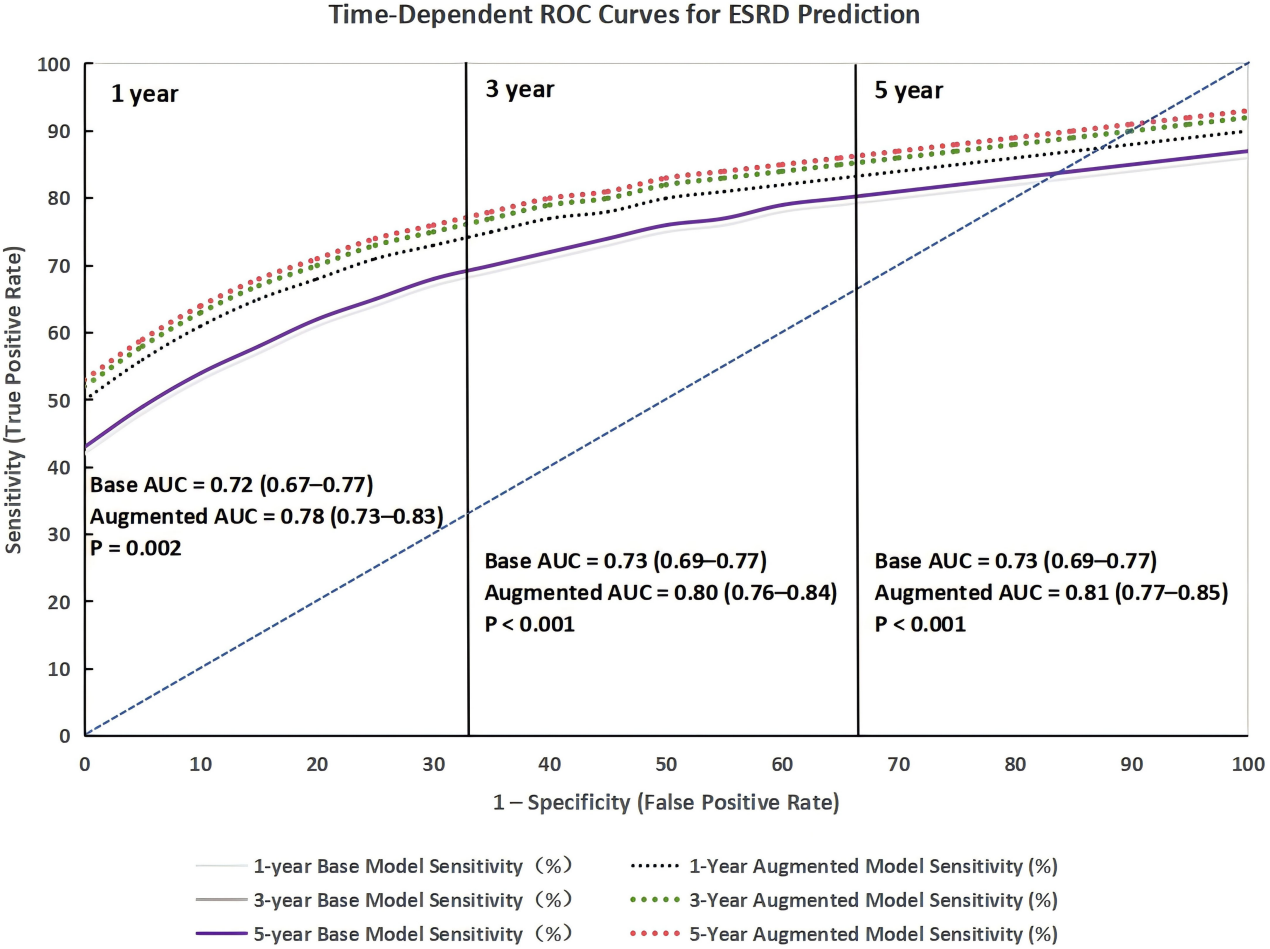
Time-dependent receiver operating characteristic (ROC) curves for ESRD prediction at 1-, 3-, and 5-year follow-up. Curves compare the base model (traditional risk factors) and augmented model (base model + 24-hour DBP average real variability [ARV]). Numbers in parentheses denote area under the curve (AUC) with 95% CIs; P-values reflect AUC differences between models (DeLong test).

**Figure 5** presents the time-dependent ROC curves for ESRD prediction at 1-year, 3-year, and 5-year follow-up. The augmented model (base model + 24-hour DBP ARV) consistently outperformed the base model at all time points: at 1-year follow-up, the AUC increased from 0.72 (95% CI 0.67–0.77) to 0.78 (95% CI 0.73–0.83, *P* = 0.002); at 3-year follow-up, the AUC increased from 0.73 (95% CI 0.69–0.77) to 0.80 (95% CI 0.76–0.84, *P* < 0.001); and at 5-year follow-up, the AUC increased from 0.73 (95% CI 0.69–0.77) to 0.81 (95% CI 0.77–0.85, *P* < 0.001). This consistent improvement over time confirms that 24-hour DBP ARV enhances long-term risk prediction in DKD.

**Figure 6:**
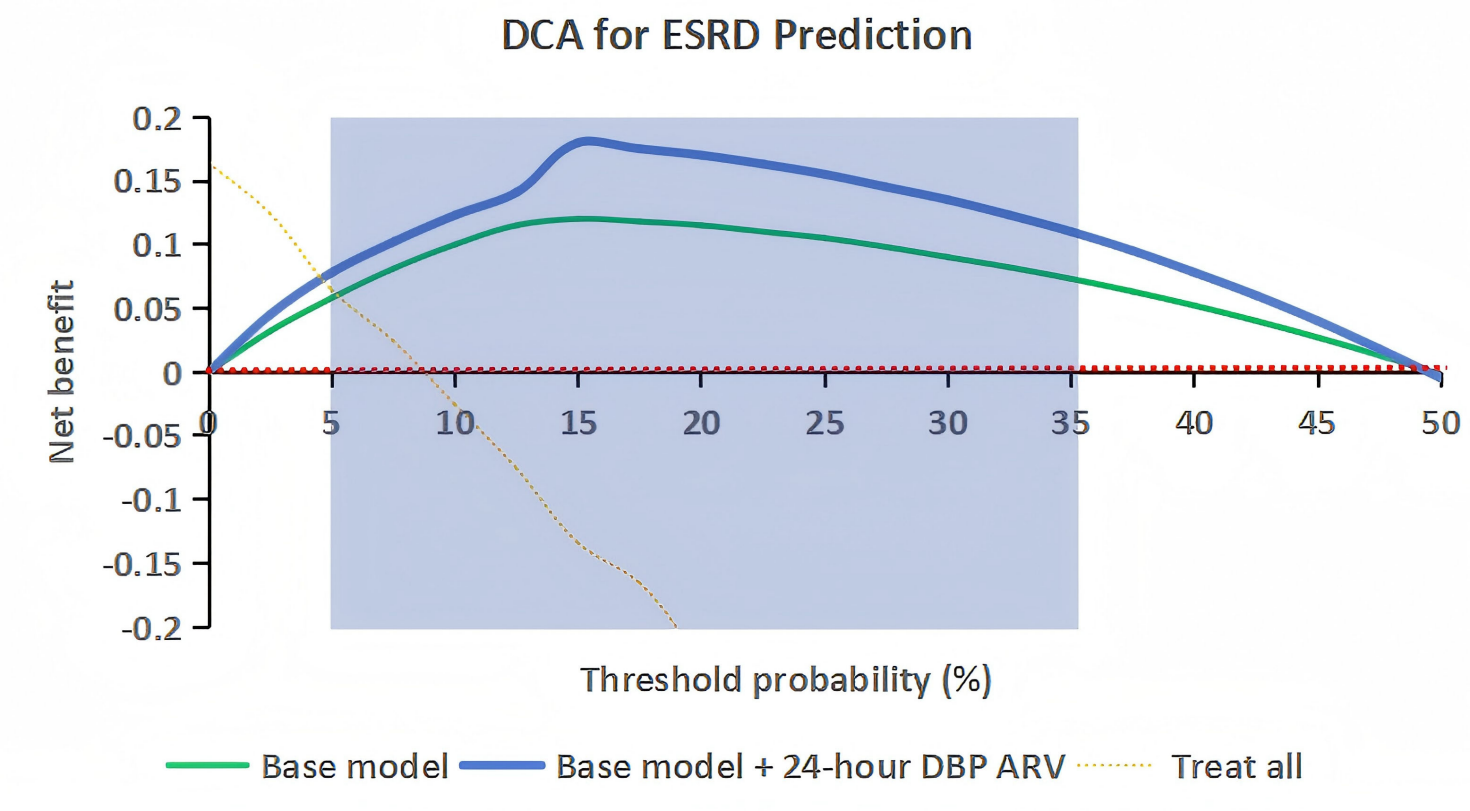
Decision curve analysis for ESRD prediction. Curves represent net benefit of the base model, augmented model (base + 24-hour DBP ARV), and “Treat all” (no risk stratification). Shaded area indicates the clinically relevant threshold range (5–35%) for DKD management.

**Figure 6** presents the decision curve for ESRD prediction, comparing the base model and the augmented model (base model + 24-hour DBP ARV). The augmented model had a higher net benefit than the base model across a threshold probability range of 5–35%, a range that is clinically relevant for DKD management given that thresholds in this interval are commonly used to initiate intensified therapy in high-risk patients. For example, at a threshold probability of 15%, the net benefit of the augmented model was 0.18, compared to 0.12 for the base model, indicating that using the augmented model to guide clinical decisions would result in more correct identifications of high-risk patients and fewer unnecessary interventions for low-risk patients.

### 3.6 Effect modification

**Figure 7:**
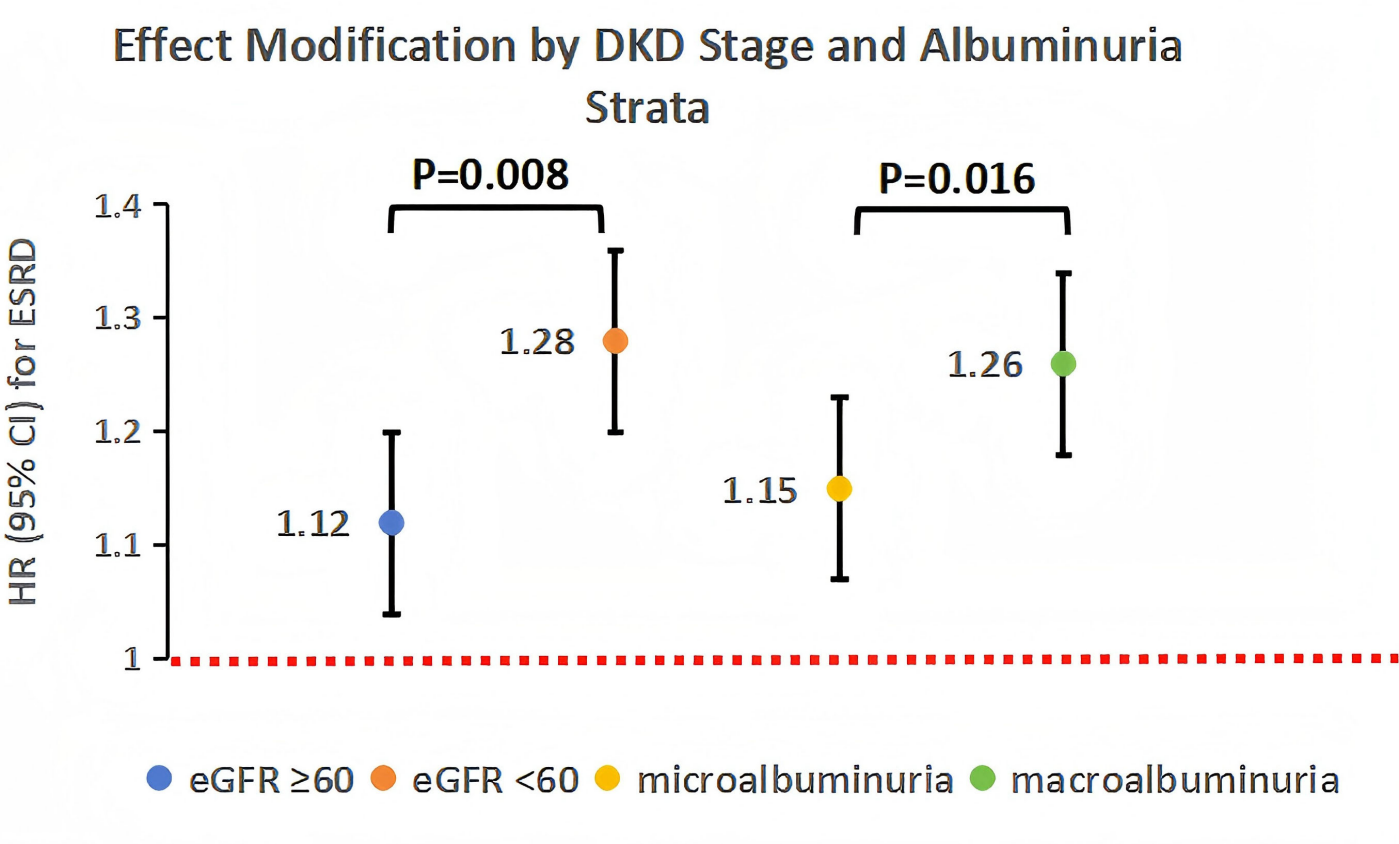
Forest plots of effect modification on the DBPV-ESRD association. **(A)** Stratified by DKD stage (eGFR ≥60 vs. <60 mL/min/1.73m^2^); **(B)** stratified by albuminuria (moderately increased albuminuria: 30–299 mg/g vs. severely increased albuminuria: ≥300 mg/g). HRs (per 1 mmHg increase in 24-hour DBP ARV) and 95% CIs were derived from multivariable Cox regression. Interaction P-values are shown for each stratification factor.

The association between 24-hour DBP ARV and ESRD was significantly modified by DKD stage (P-interaction=0.008). In stratified analyses, the association was stronger in patients with baseline eGFR <60 mL/min/1.73m^2^ (HR 1.28, 95% CI 1.20–1.36, *P* < 0.001) compared to those with eGFR ≥60 mL/min/1.73m^2^ (HR 1.12, 95% CI 1.04–1.20, *P* = 0.003; **Figure 7**). This finding suggests that patients with advanced DKD are more vulnerable to the adverse effects of DBPV, potentially due to more severe autoregulatory impairment and renal structural damage.

Albuminuria strata also modified the association between 24-hour DBP ARV and ESRD (*P*-interaction=0.016). The association was stronger in patients with severely increased albuminuria (UACR ≥300 mg/g; HR 1.26, 95% CI 1.18–1.34, *P* < 0.001) compared to those with moderately increased albuminuria (UACR 30–299 mg/g; HR 1.15, 95% CI 1.07–1.23, *P* = 0.001). severely increased albuminuria is a marker of severe glomerular damage, which may increase susceptibility to hemodynamic stress from DBPV, explaining the stronger association in this group.

No significant effect modification was observed for diabetes type (*P*-interaction=0.68) or RAAS inhibitor use (P-interaction=0.52). The association between 24-hour DBP ARV and ESRD was similar in patients with type 1 and type 2 diabetes (HR 1.20 vs. 1.23, respectively) and in patients using and not using RAAS inhibitors (HR 1.21 vs. 1.24, respectively), suggesting that the predictive value of DBPV is consistent across diabetes types and regardless of RAAS inhibitor therapy.

The interaction between 24-hour DBP ARV and 24-hour SBP ARV on ESRD risk was not significant (*P*-interaction=0.23), but patients with both 24-hour DBP ARV >10.2 mmHg and 24-hour SBP ARV >12.5 mmHg (optimal threshold for SBP ARV, derived from ROC) had a 4.5-fold higher ESRD risk than those with both parameters below thresholds (HR 4.50, 95% CI 3.61–5.62, *P* < 0.001), indicating additive risk of combined high DBPV and SBPV.

### 3.7 Sensitivity analyses

All sensitivity analyses confirmed the robustness of the study findings (**Table 7**). Excluding 156 patients with a history of baseline AKI did not alter the association between 24-hour DBP ARV and ESRD (HR 1.23, 95% CI 1.16–1.30, *P* < 0.001), nor did excluding 428 beta-blocker users (HR 1.21, 95% CI 1.14–1.28, *P* < 0.001), confirming that beta-blocker use did not confound the results. Using an alternative threshold of ≥7.5 mL/min/1.73m^2^/year for rapid eGFR decline yielded a similar association with 24-hour DBP ARV (OR 1.21, 95% CI 1.15–1.27, *P* < 0.001). After adjusting for 24-hour SBP ARV, the association between 24-hour DBP ARV and ESRD remained significant (HR 1.19, 95% CI 1.12–1.26, *P* < 0.001). Restricting the analysis to 892 patients with biopsy-proven DKD yielded a comparable association (HR 1.24, 95% CI 1.15–1.33, *P* < 0.001), confirming that the findings are not due to DKD misclassification. Adjusting for 24-hour urinary sodium excretion and MPR did not significantly change the association between 24-hour DBP ARV and ESRD (HR 1.20, 95% CI 1.13–1.27, *P* < 0.001), indicating that residual confounding from these factors is minimal (**Table 8**). Internal validation using 1,000 Bootstrap samples showed no evidence of overfitting, with the median C-statistic for the augmented model being 0.79 (95% CI 0.75–0.83), the median NRI 0.27 (95% CI 0.20–0.34), and the median IDI 0.04 (95% CI 0.03–0.06).

**Table 7:** Sensitivity analyses: 24-hour DBP ARV vs. renal outcomes.

Analysis type	Adjustment/exclusion	Outcome	Key stats
Exclude baseline AKI	n=156 (AKI within 3 months pre-ABPM)	ESRD	HR=1.23 (1.16–1.30), *P* < 0.001
Exclude beta-blocker users	n=428 (regular beta-blocker use)	ESRD	HR=1.21 (1.14–1.28), *P* < 0.001
Alternative rapid eGFR decline	≥7.5 mL/min/1.73m^2^/year (vs. ≥5)	Rapid eGFR decline	OR=1.21 (1.15–1.27), *P* < 0.001
Adjust for 24-hour SBP ARV	Add SBP ARV to model	ESRD	HR=1.19 (1.12–1.26), *P* < 0.001
Restrict to biopsy-proven DKD	n=892 (histologically confirmed)	ESRD	HR=1.24 (1.15–1.33), *P* < 0.001
Bootstrap validation	1,000 resamples (augmented model)	Predictive performance	C-stat=0.79 (0.75–0.83); NRI = 0.27 (0.20–0.34); IDI = 0.04 (0.03–0.06)

DBP ARV, diastolic blood pressure average real variability; AKI, acute kidney injury; SBP ARV, systolic blood pressure average real variability; eGFR, estimated glomerular filtration rate; ESRD, end-stage renal disease; OR/HR, odds/hazard ratio; CI, confidence interval; NRI/IDI, net reclassification/integrated discrimination improvement. All P-values <0.001.

**Table 8:** Sensitivity analysis adjusting for 24-hour urinary sodium and MPR.

Outcome	24-hour DBP ARV (per 1 mmHg increase)	95% CI	*P*-value
Rapid eGFR decline	OR=1.17	1.12–1.22	<0.001
ESRD	HR=1.20	1.13–1.27	<0.001
Composite renal events	HR=1.19	1.13–1.25	<0.001
Annual UACR increase	β=7.9 mg/g/year	5.4–10.4	<0.001

Models adjusted for Model 3 covariates + 24-hour urinary sodium excretion + medication possession ratio (MPR).

## 4 Discussion

This single-center retrospective cohort study provides hypothesis-generating evidence that 24-hour diastolic blood pressure average real variability (DBPV ARV) is an independent predictor of renal progression in patients with diabetic kidney disease (DKD), with an internally derived hypothesis-generating threshold of 10.2 mmHg for ESRD risk stratification in this study population. Dynamic increases in DBPV over time are associated with a significantly elevated risk of ESRD and rapid eGFR decline, and calcium channel blockers (CCBs) show an association with lower DBP ARV compared with RAAS inhibitors or beta-blockers alone in this cohort. Additionally, DBPV ARV adds incremental predictive value for ESRD beyond traditional risk factors, with the association between DBPV and renal outcomes being stronger in patients with advanced DKD or severely increased albuminuria. These findings highlight the potential role of ABPM-derived DBPV in DKD risk stratification, but all conclusions are limited to this single-center cohort and require rigorous validation in external multi-center, multi-ethnic populations before any clinical application is considered.

The observed association between DBPV and renal progression in this cohort is supported by well-characterized pathophysiological mechanisms linking DBP fluctuations to renal microcirculatory injury (7, 8). Diastolic BP is a key determinant of renal perfusion during ventricular relaxation, and in DKD, renal vascular autoregulatory impairment (due to arteriolar hyalinosis, endothelial dysfunction, and podocyte loss) renders the kidney vulnerable to DBP fluctuations (8, 9). Transient DBP spikes increase intraglomerular pressure and induce podocyte foot process effacement and glomerulosclerosis, while nocturnal DBP dips reduce renal blood flow and trigger ischemic tubular injury and fibrosis (9, 19). AGE formation and oxidative stress in DKD further amplify vascular reactivity to hemodynamic changes, creating a vicious cycle of DBPV-induced endothelial damage and increased BP variability (20–22). Additionally, higher DBPV was associated with elevated hsCRP in this cohort, consistent with prior evidence that BPV-induced mechanical stress activates renal inflammatory pathways (e.g., TNF-α, IL-6) and promotes interstitial fibrosis (23–25). Notably, 24-hour DBP ARV emerged as the most predictive DBPV parameter in this study, which aligns with prior work in hypertension showing that ARV— a dynamic metric capturing sequential BP changes—better reflects hemodynamic insult to target organs than static metrics (e.g., SD, CV) (10, 11). This finding underscores the importance of assessing dynamic BP fluctuations in DKD, but further studies are needed to confirm the superiority of ARV in external cohorts.

In this cohort, ROC/Youden index analysis derived an internally derived hypothesis-generating 24-hour DBP ARV threshold of 10.2 mmHg for ESRD prediction, with patients above this threshold having a 3.1-fold higher ESRD risk. This threshold is a hypothesis-generating finding only, as it was derived and validated solely within this retrospective cohort with no external validation— a critical limitation that precludes any clinical adoption at this stage (26). Dynamic DBPV changes also emerged as an important predictor of renal outcomes in this study: patients with annual DBPV increases of >1 mmHg/year had a 2.4-fold higher ESRD risk compared with those with DBPV decreases. This finding suggests that DBPV may be a modifiable parameter in DKD, but this is a purely observational association and no causal inference can be made—interventional studies are required to determine whether reducing DBPV (e.g., via pharmacological or lifestyle interventions) slows renal progression in DKD (27, 28). Additionally, nocturnal non-dipping was associated with a 76% higher ESRD risk in this cohort, consistent with prior evidence that disrupted circadian BP rhythms impair renal repair and waste clearance (12, 13). The combined elevation of DBPV and SBPV was associated with a 4.5-fold higher ESRD risk, highlighting the additive effect of systolic and diastolic BP variability on renal outcomes—a finding that requires further validation in external studies (5, 6).

In this single cohort, CCBs were associated with lower 24-hour DBP ARV compared with RAAS inhibitors or beta-blockers alone, with no significant difference between CCB monotherapy and RAAS inhibitor + CCB combination therapy. This finding is consistent with prior work showing that long-acting CCBs reduce BP variability by stabilizing vascular tone and improving endothelial function (28, 29), but it is important to emphasize that this is an observational association and not evidence of a causal effect. RAAS inhibitors are the first-line antihypertensive agents for DKD per KDIGO guidelines due to their renoprotective effects (3), and the current study does not challenge this recommendation— it merely identifies an association between CCB use and lower DBPV in this cohort. Future prospective studies are needed to evaluate whether CCB-based combination therapy reduces DBPV and improves renal outcomes in DKD, and to determine the optimal antihypertensive regimen for patients with high DBPV (29, 30).

### 4.1 Study strengths

This study has several strengths that support its hypothesis-generating findings: it is a large single-center cohort (2,143 patients) with a long median follow-up (4.8 years) and sufficient renal outcome events to power statistical analyses; it comprehensively evaluates multiple DBPV parameters (ARV, SD, CV, nocturnal dipping) and dynamic DBPV changes over time; it uses rigorous statistical methods (multivariable regression, linear mixed-effects models, DCA) and multiple sensitivity analyses to minimize confounding; it assesses the incremental predictive value of DBPV beyond traditional risk factors; and it explores DBPV differences across antihypertensive medication classes and effect modification by DKD stage/albuminuria status (18, 19). Additionally, the study uses validated ABPM devices and strict inclusion/exclusion criteria to ensure data quality (15, 16), and includes a large subset of patients with serial ABPM measurements to assess dynamic DBPV changes (17).

### 4.2 Key limitations

This study has major limitations that preclude clinical application of its findings and emphasize the need for external validation. First and foremost, it is a single-center retrospective cohort study conducted at a single tertiary hospital in Shenzhen, China. Clinical practice patterns (e.g., ABPM indications, antihypertensive medication use), patient demographics, and disease characteristics may differ significantly in other regions (e.g., other parts of China, Europe, North America) and healthcare settings (e.g., primary care, community hospitals), which severely limits the generalizability of the findings (26, 31). Second, the 10.2 mmHg DBP ARV threshold is an internally derived hypothesis-generating value with only Bootstrap internal validation and no external validation in an independent cohort— this is insufficient to support any clinical risk stratification or intervention (26). Third, the study is observational, and thus cannot establish causal relationships: all associations (e.g., DBPV with renal progression, CCB use with lower DBPV) are purely correlational, and residual confounding by unmeasured variables (e.g., sodium intake, physical activity, medication adherence, social determinants of health) cannot be ruled out (31). Fourth, DKD diagnosis included both biopsy-proven and clinically diagnosed patients, and despite exclusion of other kidney diseases and a sensitivity analysis restricted to biopsy-proven DKD, diagnostic misclassification remains a potential risk (3, 31). Fifth, serial ABPM was performed only in patients with clinically indicated BP monitoring, which may introduce selection bias in the dynamic DBPV analysis (17). Sixth, the study did not measure direct mechanistic endpoints (e.g., intraglomerular pressure, oxidative stress markers, renal fibrosis biomarkers), which limits insights into the precise molecular pathways linking DBPV to renal injury (19, 22). In addition, lack of data on specific antihypertensive drug formulations and their trough-to-peak ratios may limit interpretation of the association between CCB use and lower DBPV, as different drugs within the same class may have varying effects on BP variability. This represents a potential confounding factor that warrants consideration in future studies. Finally, the study population is predominantly Chinese, and thus the findings cannot be generalized to other ethnic or racial groups (31, 32).

### 4.3 Future research directions

Future research should focus on external validation of the findings in multi-center, multi-ethnic DKD cohorts to assess the generalizability of the DBPV ARV threshold and the association between DBPV and renal progression. Prospective randomized controlled trials (RCTs) are urgently needed to determine whether targeted interventions to reduce DBPV (e.g., long-acting CCBs, bedtime antihypertensive dosing, sodium restriction) slow renal progression in DKD (29, 30). Additionally, future studies should: (1) evaluate the association between DBPV and renal outcomes in early DKD to identify opportunities for early risk stratification; (2) measure mechanistic endpoints to elucidate the molecular pathways linking DBPV to renal injury; (3) explore the association between DBPV and other DKD outcomes (e.g., cardiovascular events, mortality); (4) develop simplified DBPV metrics using office BP or wearable devices to improve accessibility in resource-limited settings; and (5) assess the interaction between DBPV and other DKD risk factors (e.g., glycemic control, albuminuria, SGLT2 inhibitor use) (3, 29, 32, 33).

## 5 Conclusions

In this single-center retrospective cohort study, 24-hour diastolic blood pressure average real variability (DBPV ARV) is an independent predictor of renal progression in diabetic kidney disease (DKD), with an internally derived hypothesis-generating threshold of 10.2 mmHg for ESRD risk stratification. Dynamic increases in DBPV over time are associated with elevated renal risk, and calcium channel blockers (CCBs) show an association with lower DBP ARV compared with RAAS inhibitors or beta-blockers alone in this study population. Adding ABPM-derived DBPV ARV to traditional risk models improves ESRD prediction in this cohort, with the association between DBPV and renal outcomes being stronger in patients with advanced DKD or severely increased albuminuria. All findings are hypothesis-generating only and limited by the single-center, retrospective, observational design of the study— rigorous external validation in multi-center cohorts and prospective interventional studies are required before any clinical application is considered. Incorporating DBPV into DKD risk stratification is a promising area of research, but further evidence is needed to support its clinical utility.

## Data Availability

The datasets generated and/or analyzed during the current study are not publicly available due to institutional restrictions on patient data privacy but are available from the corresponding author upon reasonable request. Requests must include a detailed research proposal and approval from the requesting institution’s ethics committee to ensure compliance with data protection regulations. Requests to access the datasets should be directed to wuyang@sysush.com.

## References

[1] GBD 2021 Risk Factors Collaborators . Global burden and strength of evidence for 88 risk factors in 204 countries and 811 subnational locations, 1990-2021: a systematic analysis for the Global Burden of Disease Study 2021. Lancet. (2024) 403:2162–203. doi: 10.1016/S0140-6736(24)00933-4. PMID: 38762324 PMC11120204

[2] Alicic RZ Rooney MT Tuttle KR . Diabetic kidney disease: challenges, progress, and possibilities. Clin J Am Soc Nephrol. (2017) 12:2032–45. doi: 10.2215/CJN.11491116. PMID: 28522654 PMC5718284

[3] Kidney Disease: Improving Global Outcomes (KDIGO) Diabetes Work Group . KDIGO 2022 clinical practice guideline for diabetes management in chronic kidney disease. Kidney Int. (2022) 102:S1–S127. doi: 10.1016/j.kint.2022.06.008. PMID: 36272764

[4] Kulkarni S Parati G Bangalore S Bilo G Kim BJ Kario K . Blood pressure variability: a review. J Hypertens. (2025) 43:929–38. doi: 10.1097/HJH.0000000000003994. PMID: 40084481 PMC12052075

[5] Bae EH Lim SY Han KD Oh TR Choi HS Kim CS . Association between systolic and diastolic blood pressure variability and the risk of end-stage renal disease. Hypertension. (2019) 74:880–7. doi: 10.1161/HYPERTENSIONAHA.119.13422. PMID: 31422691 PMC6756299

[6] Chang TI Tabada GH Yang J Tan TC Go AS . Visit-to-visit variability of blood pressure and death, end-stage renal disease, and cardiovascular events in patients with chronic kidney disease. J Hypertens. (2016) 34:244–52. doi: 10.1097/HJH.0000000000000779. PMID: 26599220 PMC4818097

[7] Hall JE . Regulation of renal hemodynamics. Int Rev Physiol. (1982) 26:243–321. 6921176

[8] Ricciardi CA Gnudi L . Kidney disease in diabetes: from mechanisms to clinical presentation and treatment strategies. Metabolism. (2021) 124:154890. doi: 10.1016/j.metabol.2021.154890. PMID: 34560098

[9] Mallamaci F Tripepi G . Blood pressure variability in chronic kidney disease patients. Blood Purif. (2013) 36:58–62. doi: 10.1159/000351004. PMID: 23735729

[10] Sahutoglu T Sakaci T . Diastolic blood pressure variability in 24 hour-ABPM and outcomes of chronic kidney disease. Clin Nephrol. (2018) 90:46–52. doi: 10.5414/CN109311. PMID: 29633704

[11] Cho SMJ Lee H Yoo TH Jhee JH Park S Kim HC . Association between nocturnal blood pressure dipping and chronic kidney disease among patients with controlled office blood pressure. Am J Hypertens. (2021) 34:821–30. doi: 10.1093/ajh/hpab031. PMID: 33558892

[12] Jeong JH Fonkoue IT Quyyumi AA DaCosta D Park J . Nocturnal blood pressure is associated with sympathetic nerve activity in patients with chronic kidney disease. Physiol Rep. (2020) 8:e14602. doi: 10.14814/phy2.14602. PMID: 33112490 PMC7592496

[13] Okada H Fukui M Tanaka M Matsumoto S Mineoka Y Nakanishi N . Visit-to-visit blood pressure variability is a novel risk factor for the development and progression of diabetic nephropathy in patients with type 2 diabetes. Diabetes Care. (2013) 36:1908–12. doi: 10.2337/dc12-2087. PMID: 23340892 PMC3687293

[14] Writing Group for the CKD Prognosis Consortium Grams ME Coresh J Matsushita K Ballew SH Sang Y . Estimated glomerular filtration rate, albuminuria, and adverse outcomes: an individual-participant data meta-analysis. JAMA. (2023) 330:1266–77. doi: 10.1001/jama.2023.17002. PMID: 37787795 PMC10548311

[15] Kario K Hoshide S Chia YC Buranakitjaroen P Siddique S Shin J . Guidance on ambulatory blood pressure monitoring: a statement from the HOPE Asia Network. J Clin Hypertens (Greenwich). (2021) 23:411–21. doi: 10.1111/jch.14128. PMID: 33319412 PMC8029567

[16] Lam WY Fresco P . Medication adherence measures: an overview. BioMed Res Int. (2015) 2015:217047. doi: 10.1155/2015/217047. PMID: 26539470 PMC4619779

[17] Parati G Stergiou G O'Brien E Asmar R Beilin L Bilo G . European Society of Hypertension practice guidelines for ambulatory blood pressure monitoring. J Hypertens. (2014) 32:1359–66. doi: 10.1097/HJH.0000000000000221. PMID: 24886823

[18] Schutte AE Kollias A Stergiou GS . Blood pressure and its variability: classic and novel measurement techniques. Nat Rev Cardiol. (2022) 19:643–54. doi: 10.1038/s41569-022-00690-0. PMID: 35440738 PMC9017082

[19] Daehn IS Duffield JS . The glomerular filtration barrier: a structural target for novel kidney therapies. Nat Rev Drug Discov. (2021) 20:770–88. doi: 10.1038/s41573-021-00242-0. PMID: 34262140 PMC8278373

[20] Tabit CE Chung WB Hamburg NM Vita JA . Endothelial dysfunction in diabetes mellitus: molecular mechanisms and clinical implications. Rev Endocr Metab Disord. (2010) 11:61–74. doi: 10.1007/s11154-010-9134-4. PMID: 20186491 PMC2882637

[21] Chow EWK Fan Y Wu H Lau ESH Yang A Chow E . Age-specific associations between blood pressure and cardiovascular disease, kidney disease, and death among individuals with type 2 diabetes: a population-based cohort study. Cardiovasc Diabetol. (2026) 25:60. doi: 10.1186/s12933-025-03072-1. PMID: 41606584 PMC12922390

[22] Li J Zhang Q Li S Wang S Zhou F Zhang H . Shenhuang Liuwei Powder alleviates streptozotocin-induced diabetic ulcers in rats through the inhibition of the AGE/RAGE signaling pathway and promotion of antibacterial activity and angiogenesis via activation of the PI3K/Akt/eNOS/HIF-1α pathway. Comb Chem High Throughput Screen. (2025). doi: 10.2174/0113862073370028250326071104. PMID: 40277109

[23] Rayego-Mateos S Rodrigues-Diez RR Fernandez-Fernandez B Mora-Fernández C Marchant V Donate-Correa J . Targeting inflammation to treat diabetic kidney disease: the road to 2030. Kidney Int. (2023) 103:282–96. doi: 10.1016/j.kint.2022.10.030. PMID: 36470394

[24] Liu J Li J Zhang W Dong Y Wang F Liu J . Association analysis-based screening strategy for quality markers of Tengdan capsule in the treatment of hypertensive renal disease. Front Pharmacol. (2025) 16:1647921. doi: 10.3389/fphar.2025.1647921. PMID: 40832600 PMC12358744

[25] Wang YN Wu X Shan QY Yang Q Yu XY Yang JH . Acteoside-containing caffeic acid is bioactive functional group of antifibrotic effect by suppressing inflammation via inhibiting AHR nuclear translocation in chronic kidney disease. Acta Pharmacol Sin. (2025) 46:2975–88. doi: 10.1038/s41401-025-01598-4. PMID: 40542283 PMC12552701

[26] Moher D Liberati A Tetzlaff J Altman DG PRISMA Group . Preferred reporting items for systematic reviews and meta-analyses: the PRISMA statement. PloS Med. (2009) 6:e1000097. doi: 10.3736/jcim20090918 19621072 PMC2707599

[27] Parati G Stergiou GS Dolan E Bilo G . Blood pressure variability: clinical relevance and application. J Clin Hypertension. (2018) 20:1133–7. doi: 10.1111/jch.13304. PMID: 30003704 PMC8030809

[28] Lu Z Chen Y Li L Wang G Xue H Tang W . Combination therapy of renin-angiotensin system inhibitors plus calcium channel blockers versus other two-drug combinations for hypertension: a systematic review and meta-analysis. J Hum Hypertens. (2017) 31:1–13. doi: 10.1038/jhh.2015.125. PMID: 26740336

[29] Filippini T Malavolti M Whelton PK Naska A Orsini N Vinceti M . Blood pressure effects of sodium reduction: dose-response meta-analysis of experimental studies. Circulation. (2021) 143:1542–67. doi: 10.1161/circulationaha.121.056311. PMID: 33586450 PMC8055199

[30] Zhao X Sun J Xin S Zhang X . Study on the association between visceral adiposity index and diabetic kidney disease in hospitalized patients with type 2 diabetes mellitus in China. Front Endocrinol. (2025) 16:1549954. doi: 10.3389/fendo.2025.1549954. PMID: 40162313 PMC11951112

[31] Greenland S Pearl J Robins JM . Causal inference in epidemiology: an introduction. Int J Epidemiol. (1999) 28:1–8. doi: 10.1097/00001648-199901000-00008 10195657

[32] Wang YN Li XJ Wang WF Zou L Miao H Zhao YY . Geniposidic acid attenuates chronic tubulointerstitial nephropathy through regulation of the NF-κB/Nrf2 pathway via aryl hydrocarbon receptor signaling. Phytother Res. (2024) 38:5441–57. doi: 10.1002/ptr.8324. PMID: 39289784

[33] Młynarska E Buławska D Czarnik W Hajdys J Majchrowicz G Prusinowski F . Novel insights into diabetic kidney disease. Int J Mol Sci. (2024) 25:10222. doi: 10.3390/ijms251810222. PMID: 39337706 PMC11432709

